# METTL16 promotes glycolytic metabolism reprogramming and colorectal cancer progression

**DOI:** 10.1186/s13046-023-02732-y

**Published:** 2023-06-20

**Authors:** Wei Wei, Zhong-Yuan Zhang, Bin Shi, Yike Cai, Hou-Shun Zhang, Chun-Lei Sun, Yun-Fei Fei, Wen Zhong, Shuang Zhang, Chen Wang, Bing He, Guan-Min Jiang, Hao Wang

**Affiliations:** 1 Department of Laboratory Medicine, The First Affiliated Hospital of USTC, Division of Life Sciences and Medicine, University of Science and Technology of China, Hefei, China; 2 Core Unit of National Clinical Research Center for Laboratory Medicine, Hefei, China; 3 Department of Radiology, The First Affiliated Hospital of USTC, Division of Life Sciences and Medicine, University of Science and Technology of China, Hefei, China; 4 Department of General Surgery, The First Affiliated Hospital of USTC, Division of Life Sciences and Medicine, University of Science and Technology of China, Hefei, China; 5 Center for Certification and Evaluation, Guangdong Drug Administration, Guangzhou, China; 6 Department of Pathology, The First Affiliated Hospital of USTC, Division of Life Sciences and Medicine, University of Science and Technology of China, Hefei, China; 7 Department of Clinical Laboratory, The Fifth Affiliated Hospital, Sun Yat-sen University, Zhuhai, China

**Keywords:** Colorectal cancer, m6A, METTL16, SOGA1, PDK4

## Abstract

**Background:**

Glycolysis is the key hallmark of cancer and maintains malignant tumor initiation and progression. The role of N6-methyladenosine (m6A) modification in glycolysis is largely unknown. This study explored the biological function of m6A methyltransferase METTL16 in glycolytic metabolism and revealed a new mechanism for the progression of Colorectal cancer (CRC).

**Methods:**

The expression and prognostic value of METTL16 was evaluated using bioinformatics and immunohistochemistry (IHC) assays. The biological functions of METTL16 in CRC progression was analyzed in vivo and in vitro. Glycolytic metabolism assays were used to verify the biological function of METTL16 and Suppressor of glucose by autophagy (SOGA1). The protein/RNA stability, RNA immunoprecipitation (RIP), Co-immunoprecipitation (Co-IP) and RNA pull-down assays were used to explore the potential molecular mechanisms.

**Results:**

SOGA1 is a direct downstream target of METTL16 and involved in METTL16 mediated glycolysis and CRC progression. METTL16 significantly enhances SOGA1 expression and mRNA stability via binding the “reader” protein insulin-like growth factor 2 mRNA binding protein 1 (IGF2BP1). Subsequently, SOGA1 promotes AMP-activated protein kinase (AMPK) complex ubiquitination, inhibits its expression and phosphorylation, thus upregulates pyruvate dehydrogenase kinase 4 (PDK4), a crucial protein controlling glucose metabolism. Moreover, Yin Yang 1 (YY1) can transcriptionally inhibit the expression of METTL16 in CRC cells by directly binding to its promoter. Clinical data showed that METTL16 expression is positively correlated to SOGA1 and PDK4, and is associated with poor prognosis of CRC patients.

**Conclusions:**

Our findings suggest that METTL16/SOGA1/PDK4 axis might be promising therapeutic targets for CRC.

**Supplementary Information:**

The online version contains supplementary material available at 10.1186/s13046-023-02732-y.

## Background

Colorectal cancer (CRC) is the fourth most common human malignancy and third leading cause of cancer-related deaths worldwide [1]. CRC is a multifactorial disease involving genetic, hereditary, environmental and lifestyle risk factors [2]. Most CRC cases are sporadic and sequential accumulation of mutations in Wnt, EGFR, P53, TGF-β signaling pathways, APC, KRAS, BRAF and DNA mismatch repair genes has been found to lead to initiation and progression of CRC [2]. Beyond these genetic events, exhaustive transcriptomic analyses have revealed a consensus molecular classification of CRC with four consensus molecular subtypes based on unique clonal, stromal and immune dependencies. These consensus molecular subtypes recapitulate the heterogeneity observed in CRC and predict the response to targeted therapies [3]. All this greatly contributed to the improvement in the diagnosis and treatment of CRCs. However, especially at advanced stages, CRC become difficult to treat, making metastasis with poor prognosis due to the lack of early diagnostic and effective intervention. These poor treatment outcomes highlight the need to better understand the mechanisms that account for CRC initiation, progression and spreading.

As a prominent hallmark of cancer, energy metabolism reprogramming greatly supports the initiation and progression of malignant tumors [4, 5]. Aerobic glycolysis, the main characteristic of energy metabolism reprogramming in tumors, enhances glycolysis activation and preferential lactate fermentation even in the presence of oxygen, which is referred to as the Warburg effect [6, 7]. Cancer cells maintain a sustained proliferation and metastatic phenotype that is dependent on the energy and biosynthesis generated by aerobic glycolysis [8]. Moreover, aerobic glycolysis plays an important role in the regulation of aggressive tumor microenvironments, such as proinflammatory factor secretion, angiogenesis, and immune evasion [9-11]. Thus, targeting aerobic glycolysis, including glucose transporters, glycolytic enzymes, lactate production, and related signaling pathways, has been identified as an attractive therapeutic approach for cancer [12, 13]. Several specific inhibitors targeting aerobic glycolysis have shown potential therapeutic efficacy in preclinical studies, highlighting the potential role of glycolysis as a therapeutic target for cancer treatment [14]. Aerobic glycolysis could also influence the initiation and progression of CRC, including self-renewal, proliferation, metastasis and immunotolerance [2]. Notably, some of the genetic drivers of CRC are well known regulators of aerobic glycolysis, such as Wnt, KRAS and p53 [2]. Experimental research convincingly established that glycolysis and signaling pathways of CRC cells undergo a significant change that could be targeted with novel pharmacological strategies [2]. Therefore, a better understanding of the molecular basis of glycolysis in CRC is crucial to explore diagnostic biomarkers and therapeutic targets.

N6-methyladenosine (m6A) methylation, the most abundant posttranscriptional modification that ubiquitously occur in eukaryotic mRNAs, plays a critical role in the regulation of mRNA splicing, decay, stability, translation, and nuclear export [15, 16]. As a dynamic and reversible process, m6A modification is catalyzed by m6A methyltransferases [also known as writer: METTL3, METTL14, WT1-associated protein (WTAP) and METTL16] and eliminated by demethylases [Fat mass and obesity-associated protein (FTO) and AlkB homolog 5 RNA demethylase (ALKBH5)] [17]. In addition, RNA-binding proteins [YTH domain family of protein1/2/3 (YTHDF1/2/3), IGF2 mRNA binding protein1/2/3 (IGF2BP1/2/3), and Heterokaryotic nuclear RNA protein C (HNRNPC)] function to identify and bind the m6A motif to control RNA metabolism [18, 19]. Previous studies have shown that m6A modification is involved in various physiological and pathological processes, such as stem cell differentiation, DNA damage, circadian periods, embryonic development, and spermatogenesis [20, 21]. Recently, emerging evidence has demonstrated that m6A modulators play an indispensable role in tumorigenesis and malignant progression in different types of tumors [22, 23]. In CRC, multiple m6A regulators are reported to be abnormally regulated and act either as oncogenes or tumor suppressors, which play an important role in tumor occurrence and progression [24]. m6A regulators may serve as promising diagnostic biomarkers and potential therapeutic targets of CRC [24]. However, the role and molecular mechanism of m6A methyltransferase METTL16 in CRC, especially in glycolytic metabolism remains elusive.

In the present study, we demonstrated that METTL16 plays an oncogenic role in CRC. The biological role, molecular mechanism, and clinical significance of METTL16 in glycolytic metabolism and progression was revealed. Our findings suggest that METTL16 may be a novel potential prognostic biomarker and therapeutic target in CRC.

## Materials and methods

### Cell culture and transfection

The colon epithelial cell line NCM460 and CRC cell lines SW620, Lovo, and RKO were obtained from the Cell Repository of the Chinese Academy of Sciences (Shanghai) and the CRC cell lines HCT8, HCT15, HCT116, HT29, and SW480 cells were obtained from the American Type Culture Collection (ATCC). HCT15 and HCT8 cells were cultured in RPMI-1640 (Hyclone), HCT116 and HT29 cells were cultured in McCoys’5 A (Hyclone), and SW480, SW620, RKO and NCM460 cells were grown in DMEM (Gibco). All media were supplemented with 10% fetal bovine serum (FBS, BI). For transfection, siRNA or plasmids were transfected into cells using Lipofectamine 3000 reagent (Invitrogen Life Technology, USA) according to the manufacturer’s instructions. Stably transfected cells expressing shMETTL16 (shM16), shNC, pHBLV-con (Ctrl), pHBLV-METTL16 (OEM16), shSOGA1 and shNC were obtained from lentivirus packaging. siRNAs and their corresponding negative controls (siNC) were synthesized by GenePharma (Shanghai, China). The siRNA sequences used are listed in Table S1.

### Western blotting

Western blotting was performed as previously described [25]. Primary antibodies were listed as: anti-METTL16 (#17,676, CST), anti-SOGA1 (A16597, Abclonal), anti-IGF2BP1 (22803-1-AP, Proteintech), anti-IGF2BP2 (11601-1-AP, Proteintech), anti-IGF2BP3(14642-1-AP, Proteintech), anti-YTHDC1 (14392-1-AP, Proteintech), anti-YTHDF1 (17479-1-AP, Proteintech), anti-YTHDF2 (24744-1-AP, Proteintech), anti-YTHDF3 (25537-1-AP, Proteintech), anti-PDK4 (PB0823, BOSTER), anti-YY1 (66281-1-Ig, Proteintech), anti-CEBPB (BM3970, BOSTER), anti-pAMPK (#2535,CST), anti-AMPKα1 (#5832,CST), anti-AMPKβ1 (10308-1-AP, Proteintech), anti-AMPKγ1 (10290-1-AP, Proteintech), anti-GAPDH (60004-1-lg, Proteintech) or β-actin (66009-1-lg, Proteintech) were used as the internal control.

### Real-time quantitative reverse transcription PCR (qRT-PCR)

Total RNA of cells was extracted by TRIzol Regent (Invitrogen) according to the previous protocols followed by cDNA synthesis with PrimeScript™ RT Master Mix (RR036A, TAKARA). mRNA expression levels were measured by TB Green® Premix Ex Taq™ II Kit (RR820A, TAKAR) on Applied Biosystems StepOnePlus. Relative RNA amount of each group was calculated using the 2^–ΔΔCt^ method with normalization by GAPDH, which was used as a control for normalization. The primers used for qRT-PCR are listed in Table S2.

### Metabolic assay

The glucose and lactate concentrations in the cultured media were detected using glucose colorimetric/fluorometric assay kit (Biovision) and lactate colorimetric/fluorometric assay kit (Biovision) according to the manufacturer’s instructions, respectively. All samples were measured in triplicates.

### ECAR and OCR

The extracellular acidification rate (ECAR) and oxygen consumption rate (OCR) were analyzed using a Seahorse XF96 instrument (Seahorse Bioscience, USA). For OCR test, cell medium was replaced with assay medium (Seahorse Bioscience) supplemented with 1 mM pyruvate, 10 mM glucose, and 2 mM glutamine for 1.5 h at 37 °C, then measured using the XF Cell Mito Stress Kit (Seahorse Bioscience). The concentrations of oligomycin, FCCP and rotenone/antimycinA were 1.0 µM, 1.0µM and 0.5µM, respectively. For ECAR test, cells were incubated in the assay medium (Seahorse Bioscience) with 1 mM glutamine for 1.5 h at 37 °C, then measured using the Glycolytic Stress Test Kit (Seahorse Bioscience). The concentrations of glucose, oligomycin, and 2-DG were 10mM, 1µM and 50mM, respectively. The OCR and ECAR results were adjusted using Seahorse XF96 Wave software.

### RNA stability and protein stability assays

Cells were treated with 5 µg/ml actinomycin D (Act-D) or 100 µg/ml cycloheximide (CHX) the indicated time periods for RNA and protein stability assays, respectively. For the RNA stability assay, cells were extracted total RNA and analyzed by qRT-PCR. For protein stability assays, cells were lysed and analyzed by western blotting.

### Co-immunoprecipitation (Co-IP) assay

Cells were lysed using IP buffer supplemented with protease inhibitors. Cell lysate was pre-cleaned with 20 µl DynabeadTM Protein A (10001D, ThermoFisher Scientific) for 2 h at 4 °C. Pre-cleaned cell lysate was further incubated with antibody or IgG at 4 °C overnight. Then 20 µl washed magnetic beads were added to each reaction and incubated for 2 h at 4 °C。The co-precipitated complex was washed with IP buffer and boiled in SDS loading buffer. The eluted samples were analyzed by western blotting.

### Nuclear/Cytoplasm separation

Nuclear and cytoplasm fractions from SW620 cells (1 × 10^6^) were obtained by using a nuclear/cytosol fractionation kit (BioVision, Milpitas, CA, USA) following the manufacturer’s guidelines. The β-actin gene was used as a cytoplasmic localization control and LaminB1 gene was used as a nucleus localization control.

### CRC tissue specimens and clinical data

86 CRC and corresponding adjacent normal tissue samples were collected at the First Affiliated Hospital of the USTC from January 2015 to December 2016. Informed consent was obtained from each patient before our study and those who received systemic or local therapy were not included in the present study. This research was authorized by the First Affiliated Hospital of the USTC Ethics Committee. The clinicopathological characteristics of these CRC patients are presented in Table S3.

### Immunohistochemistry (IHC) staining

IHC was performed to detect the expression of target proteins according to our previous study [25]. Briefly, following deparaffinization, rehydration and antigen retrieval, CRC and adjacent normal tissue sections were conjugated with primary antibodies at 4 °C overnight. After incubation with secondary and development of Diaminobenzidine (DAB), the staining scores of target proteins were evaluated blindly by two independent pathologists by multiplication of the staining intensity grade (0, 1, 2 or 3 indicated negative, weak, moderate or strong stains, respectively) and proportion of positive stains (0, 1, 2, 3 or 4 implied positive areas of 0–5%, 6–25%, 26–50%, 51–75% or 76–100%, respectively).

### Invasion and migration

Invasion and migration were performed to detect the expression of target proteins according to our previous study [25]. Briefly, 5 × 10^4^ cells in 200 µl serum-starved medium were seeded into the upper chamber (8.0 μm pore size filter, Corning) with or without coated Matrigel (BD, Bioscience), while 600 µl medium containing 10% FBS was placed into the lower chamber in 24-well plates. After incubation in 37 °C for 48 h, cells passed through the membrane were immobilized by methyl alcohol and stained with 0.2% crystal violet solution. Subsequently, the penetrated cells were photographed and calculated under Olympus microscope.

### MeRIP-sequencing

MeRIP-sequencing and data analysis were supported by Genesky Biotechnologies Inc (Shanghai, China). Experimental protocols were performed as described in our previous study [25]. Total RNA was extracted from SW620 cells transfected with shMETTL16-2 or shNC using TRIZOL reagent, followed by poly (A) + RNA purification and fragmentation using the NEBNext Poly (A) mRNA Magnetic Isolation Kit (New England Biolabs, UK). Concentration of RNA was detected on Nanodrop 2000 (Thermo Fisher Scientific, USA) and the integrality was guaranteed by Agilent 2100 Bioanalyzer (Agilent Technologies, USA). Dynabeads Protein A (Thermo Fisher Scientific, USA) was mixed with rabbit anti-m6A antibody (Synaptic system, Germany) at 4 °C for 2 h in advance, then fragmented mRNA was incubated with the mixture for another 2 h to precipitate m6A-enriched RNAs. Qualified samples underwent Library Pooling and Sequencing using Illumina HiSeq 2500 machines. Following quality filter, the raw sequence data was mapped to human genome GRCh37/hg19 utilizing the HISAT2 software (v2.0.5) and the results were subjected to analyzed bioinformatically and statistically. The peak calling data and RNA sequencing data were described in supplementary materials.

### RNA immunoprecipitation (RIP)

RIP experiments were performed as previously described [25]. Briefly, cell lysates were rotary incubated with 1 µg specific antibodies against IgG, IGF2BP1 or m6A at 4 °C overnight, then added 20 µl washed magnetic beads to each reaction and incubated at 4 °C for 2 h. After washed 3 times, the target RNAs in the immunoprecipitation complex were purified for further analysis by qRT-PCR. The relative enrichment of RNA was normalized to the input.

### RNA pull-down assay

RNA pulldown assay was performed as described in our previous study [25]. Briefly, 1 × 10^7^ cells were lysed and the lysates were rotary incubated with 3 µg biotin-labeled probe mixed with cocktail and ribonuclease inhibitor at 4 °C overnight. Then 20 µl of pre-cleared streptavidin magnetic beads (88,816, ThermoFisher Scientific) were added to the cell lysates to precipitate the RNA-protein complex. After elution from the beads with lysis buffer for 3 times, the co-precipitated proteins were boiled with loading buffer for 10 min for further analysis by western blotting. The biotin probe was designed and synthesized by GenePharma. The probe was fully complementary to the CDS specific sequence of SOGA1 mRNA. The sequence was listed as follows: AGCAGGAAGTTGTGCTTGAATTGCT, negative control: AGCAATTCAAGCACAACTTCCTGCT.

### Chromatin immunoprecipitation (ChIP) assay

The ChIP assay was performed in SW620 and SW480 cells using the SimpleChIP® Enzymatic Chromatin IP Kit (#9003, CST) according to the manufacturer’s instructions. The cells (1 × 10^7^) were crosslinked with 1% formaldehyde for 15 min at room temperature. Chromatin fragments ranging from 200 to 900 bp were generated by lysis with SDS lysis buffer, followed by sonication. After centrifugation, the supernatant from lysate was diluted with ChIP buffer and the DNA-protein complex was precipitated with anti-YY1 antibody or anti-IgG antibody (#2729, CST) at 4 °C overnight. The DNA-protein complex was incubated with 30 µl ChIP-grade Protein G magnetic beads for 2 h at 4 °C. After elution from the beads, the immunoprecipitates were de-crosslinked at 65 °C for 3 h for further analysis by qRT-PCR. The ChIP primers that were used are listed in Table S2.

### Animal experiments

5-weeks old male BALB/c athymic nude mice were obtained from the First Affiliated Hospital of the USTC Animal Center and were randomly divided into experimental and control groups. 2 × 10^6^ SW620 cells stably overexpressed or silencing METTL16 were injected into the right flank of mice to observe tumor growth. Tumor volumes were monitored once a week after injection and calculated using the formula 0.5 × a^2^ × b (a and b indicate short and long tumor diameters, respectively). Four weeks later, the mice were sacrificed, and the tumors were removed and weighed for histological analysis and further studies. This study was authorized by the First Affiliated Hospital of the USTC Ethics Committee.

### Statistical analysis

Statistical analyses were performed using GraphPad Prism 7.0 (GraphPad, Inc., USA) and SPSS 19.0 (SPSS, Inc., USA). The experiments were repeated at least three times. Data are shown as the mean ± SD by a two-tailed Student’s t test after homogeneity of variances test. One-way ANOVA was used to compare the differences among multiple comparisons. The correlation between protein expression in tissue and clinicopathological characteristics was analyzed using the chi-square test. The overall survival curve was analyzed using Kaplan–Meier analysis and the difference was measured using the log-rank test. Statistical significance was set at *p* < 0.05.

## Results

### METTL16 overexpression correlates with poor prognosis of CRC patients

**Fig. 1:**
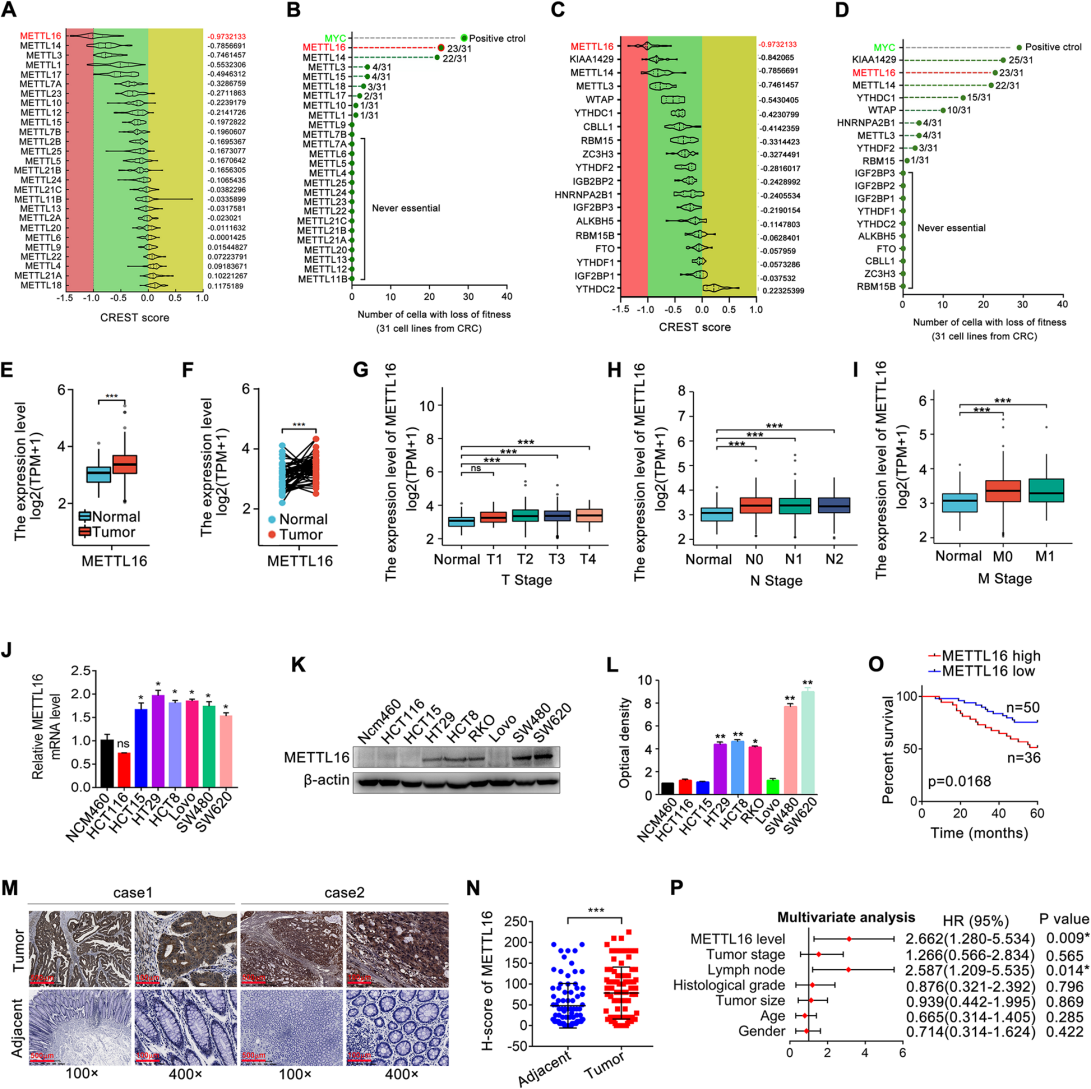
METTL16 is upregulated in CRC and correlated with poor prognosis. **A**, **C** CERES scores of a series of METTL family members and m6A regulators from CRISPR associated protein 9 knockout screening datasets across 56 human CRC cell lines, respectively. The raw data were downloaded from DepMap (https://depmap.org/portal/). CERES scores 0 and − 1 represent the median effect of non-essential genes and common core essential genes, respectively. The lower CERES score represents the higher dependency of the specific gene on cancer progression. The average of CERES scores for each METTL family member was displayed on the right. **B**, **D** Another CRISPR associated protein 9 knockout screening datasets across 31 human CRC cell lines were analyzed. The raw data were downloaded from https://score.depmap.sanger.ac.uk/. MYC was identified as the positive control, which was recognized as the promising cancer therapeutic target. The number of cells with essential function and the number of total CRC cell lines were displayed for each gene. For example, METTL16 (31/23) represents that knockout of METTL16 displays essential function in 23 of the 31 CRC cell lines. **E**-**F** METTL16 expression in the TCGA CRC cohort. **G**-**I** Association of METTL16 mRNA expression with tumor size (**G**), lymph node metastasis (**H**), and distant metastasis (**I**) in CRC patients in TCGA database. N0: no lymph node metastasis; N1: nearby lymph node metastasis; N2: distant lymph node metastasis; M0: no distant metastasis; M1: distant metastasis. (**J**-**L**) qRT-PCR (**J**) and western blotting (**K**, **L**) analysis of METTL16 mRNA and protein expression in CRC cell lines and intestinal epithelium cells Ncm460. **M** Representative images of METTL16 IHC staining in CRC tissues and adjacent tissues. **N** Scores of METTL16 IHC staining in CRC tissues and adjacent tissues. **O** Kaplan-Meier survival curves of OS based on METTL16 IHC staining in CRC patients. **P** Multivariate analysis of CRC patients based on COX regression model was displayed. Some factors associated with CRC patient clinical outcomes were introduced to the model. *P < 0.05, ***P < 0.001

Through analysis of two independent large-scale genome-wide clustered regularly interspaced short palindromic repeats–CRISPR associated protein 9 knockout screening datasets, we found that among the METTL family members, METTL16 is the most essential gene for the survival of CRC cells (Fig. 1A, B). Importantly, among the main m6A regulators, METTL16 also showed the important role in the survival of CRC (Fig. 1C, D), implying its functional significance in CRC. TCGA and GEO databases showed that METTL16 was remarkably upregulated in CRC tissues compared with normal tissues (Fig. 1E, F and Fig. S1A). Furthermore, TGGA data showed the association with METTL16 expression and clinicopathologic variables of CRC. As shown in Fig. 1G-I and Fig. S1B, increased expression of METTL16 correlated significantly with the tumor size, lymph node metastasis, distant metastasis, and clinical stage grade. The mRNA expression of METTL16 in CRC cell lines was also higher than that in normal colonic epithelial cells (Fig. 1J). Similarly, METTL16 protein expression in CRC cell lines was generally higher than that in the normal colonic epithelial cells (Fig. 1K, L). In addition, the results of immunohistochemistry (IHC) staining of CRC samples and adjacent para-cancer tissue further verified that METTL16 expression was enhanced in CRC tissues (Fig. 1M and N). Increased METTL16 protein expression is associated with poor survival in CRC patients (Fig. 1O). Moreover, multivariate Cox regression analysis showed that METTL16 protein expression may be an independent predictor of survival in CRC patients (Fig. 1P) and the ROC curve showed that METTL16 mRNA expression may be a predictor of CRC tumorigenesis (Fig. S1C). Collectively, METTL16 was upregulated in CRC and may play an important role in CRC progression.

### METTL16 promotes CRC progression

**Fig. 2:**
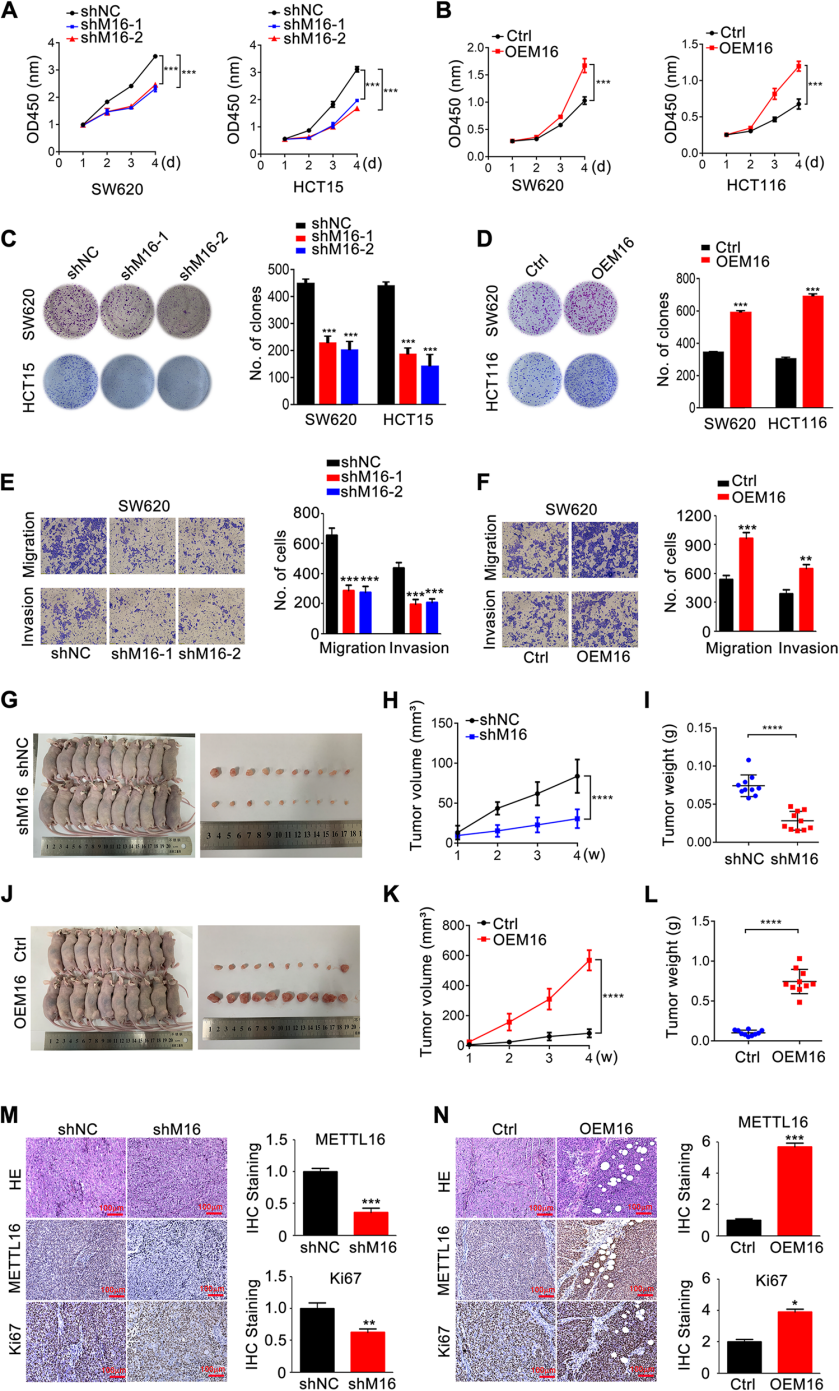
METTL16 promotes CRC progression in vitro and in vivo. **A** CCK8 assay was performed to detect the proliferation of SW620 and HCT15 cells with METTL16 knockdown (shM16). **B** CCK8 assay was performed to detect the proliferation of SW620 and HCT116 cells with METTL16 overexpression. **C**-**D** Colony formation assays were performed to detect the proliferation of SW620 and HCT15 cells with METTL16 knockdown (**C**) or SW620 and HCT116 cells with METTL16 overexpression (**D**). **E**-**F** Transwell assays were performed to detect the migrative and invasive capacity of SW620 cells with METTL16 knockdown (**E**) or overexpression (**F**). **G**, **J** Xenografts derived from SW620-shM16 or HCT116-OEM16 cells and relative controls (*n* = 10). **H**, **I**, **K**, **L** Tumor volume was recorded at indicated time to establish a growth curve (H, K) and tumors weight (**I**, **L**) were measured after mice sacrificed. **M**, **N** Hematoxylin-eosin (HE) staining and METTL16 and Ki67 IHC staining of xenografts tissues. ***P < 0.001, ****P < 0.0001

To investigate the role of METTL16 in CRC progression, the expression of METTL16 was knocked down and overexpressed in CRC cells using two shRNAs (shM16-1, shM16-2) and a pHBLV-METTL16 vector (OEM16) respectively. As shown in Fig. S2A-D, the results of qRT-PCR and western blotting confirmed that transfection of shM16-1 and shM16-2 significantly decreased METTL16 mRNA and protein expression (Fig. S2A, B), while transfection of pHBLV-METTL16 increased METTL16 mRNA and protein expression in CRC cells (Fig. S2C, D). Knockdown of METTL16 decreased the proliferation and colony formation of CRC cells, whereas overexpression of METTL16 had the opposite effect (Fig. 2A-D). Similarly, METTL16 knockdown inhibited the migrative and invasive ability of CRC cells, while METTL16 overexpression promoted CRC cells metastasis (Fig. 2E, F and Fig. S2E, F). The function of METTL116 was further evaluated in the METTL16 knockdown and overexpression CRC xenografts, which indicated that overexpression of METTL116 elevated tumor growth, whereas suppression of METTL16 inhibited tumor growth, as reflected by tumor size, volume, and weight, (Fig. 2G-L). Moreover, METTL16 knockdown decreased the expression of Ki67, a biomarker of tumor proliferation, whereas METTL16 overexpression promoted Ki67 expression in vivo (Fig. 2M, N). Taken together, these results demonstrated that METTL16 play an important role in promoting CRC progression.

#### Suppressor of glucose by autophagy (SOGA1) is a direct target of METTL16

**Fig. 3:**
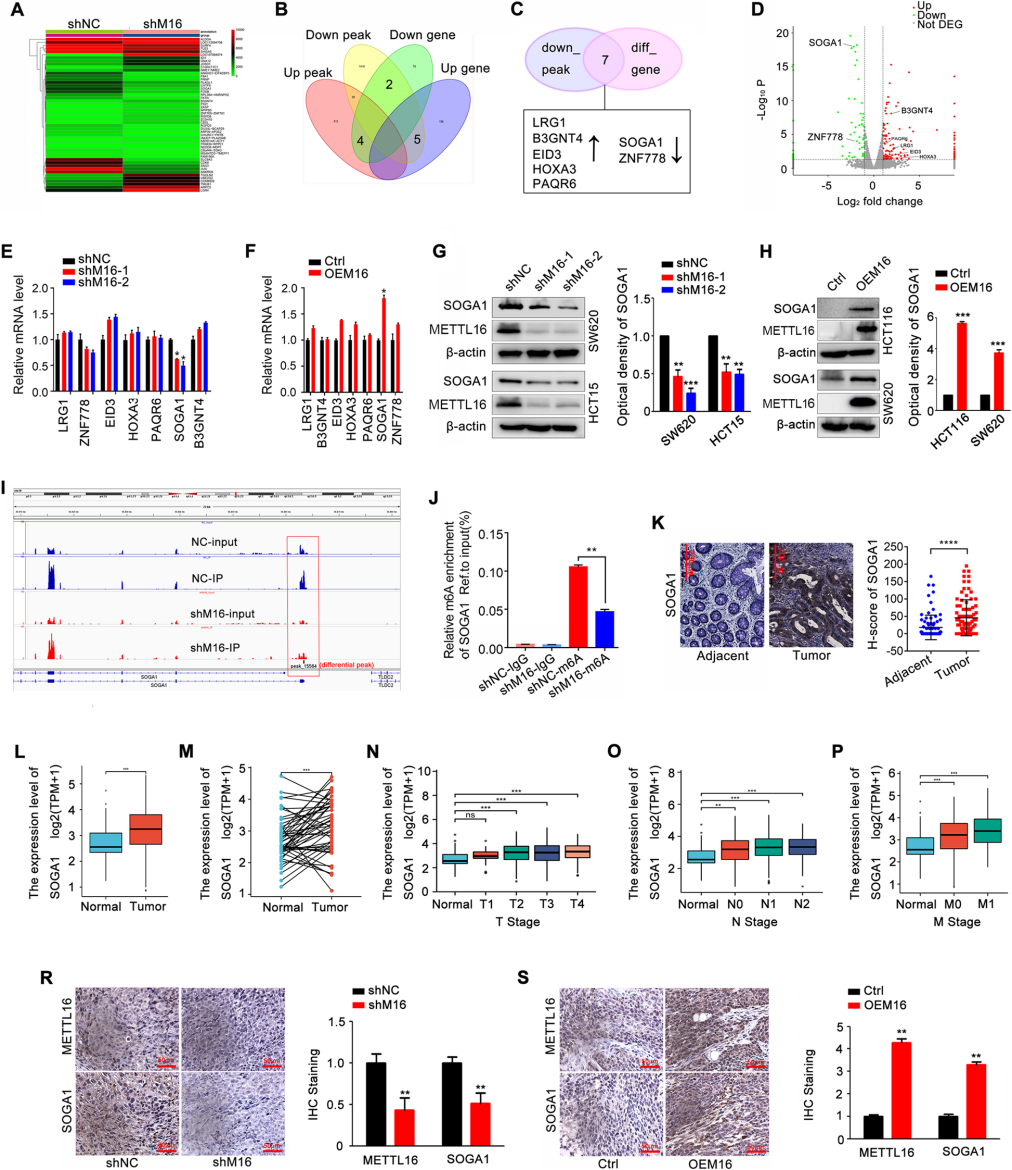
SOGA1 is identified as the direct target of METTL16. **A** Heatmap showing the expression profile of differentially methylated genes after METTL16 knockdown in SW620 cells. **B** A total of 11 differential genes were classified according to the level of mRNA and m6A peak in SW620 cells with METTL16 knockdown. **C** 7 candidate target genes of METTL16 came from the intersection of RNA-sequencing and MeRIP-sequencing. **D** Volcano plot showed the 7 candidate target genes of METTL16. **E**-**F** The 7 candidate target genes mRNA expression in SW620 cells with METTL16 knockdown or overexpression were detected by qRT-PCR. **G**, **H** The SOGA1 protein expression in SW620 and HCT15 cells with METTL16 knockdown (**G**) and in SW620 and HCT116 cells with METTL16 overexpression (**H**) were detected by western blotting. **I** The m6A peak visualization of m6A-seq in SOGA1 transcripts in SW620 cells with or without METTL16 knockdown was shown. **J** Relative m6A enrichment of SOGA1 mRNA in SW620 cells with or without METTL16 knockdown was analyzed and normalized to input by using MeRIP-qPCR. **K** Representative images of SOGA1 IHC staining (left) and scores (right) in CRC tissues and adjacent tissues. **L**, **M** SOGA1 expression in the TCGA CRC cohort. **N**-**P** Association of SOGA1 mRNA expression with tumor size (**N**), lymph node metastasis (**O**), and distant metastasis (**P**) in CRC patients in TCGA database. **R**, **S** IHC (METTL16 and SOGA1)-stained paraffin-embedded sections obtained from xenografts based on SW620 cells with METTL16 knockdown or overexpression. **P < 0.01

To explore the molecular mechanisms of METTL16-induced proliferation in CRC, MeRIP-sequencing and RNA-sequencing were performed in CRC cells with stable METTL16 knockdown and in relative control cells. MeRIP-sequencing identified 18,812 and 17,211 m6A peaks in the control and METTL16-deficient cells, respectively (Fig. S3A). These m6A peaks enriched close to the stop codons and mainly located in exon region (Fig. S3B-D). In addition, RNA-sequencing data showed that METTL16 loss upregulated 281 genes and downregulated 756 genes (Fig. S3E, F). The top 50 differential genes are shown in Fig. 3A. Nine changed genes and peaks overlapped (Fig. 3B). In the down peaks, there are seven genes expression were altered, including LRG1, B3GNT4, EID3, HOXA3, PAQR6, SOGA1, and ZNF778 (Fig. 3C, D). GO analysis of these differential genes showed that these genes were enriched in tumor-associated metabolism pathways (Fig. S3G). Through verification, knockdown of METTL16 decreased mRNA SOGA1 expression and overexpression of METTL16 upregulated mRNA SOGA1 in CRC cells (Fig. 3E, F). Similarly, METTL16 positively regulated SOGA1 protein expression (Fig. 3G, H). Importantly, in the MeRIP-seq data, we detected one m6A peak of SOGA1 mRNA, which was diminished upon METTL16 knockdown (Fig. 3I). The results of MeRIP-qPCR showed that m6A modified SOGA1 mRNA was significantly decreased when METTL16 was knocked down (Fig. 3J). A global methylation inhibitor 3-deazaadenosine (DAA) treatment dramatically decreased the SOGA1 mRNA and protein in CRC cells (Fig. S4A-D). Upon separation of nuclear and cytoplasmic proteins, SOGA1 was found to located in the cytoplasm, and suppression of METTL16 downregulated cytoplasmic SOGA1 expression in CRC cells (Fig. S4E). As an oncogene, SOGA1 protein and mRNA expression was significantly increased in CRC tissues compared with normal tissues (Fig. 3K-M). Increased expression of SOGA1 correlated significantly with the tumor size, lymph node metastasis, distant metastasis, and clinical stage grade (Fig. 3N-P and Fig. S4F). The ROC curve showed that SOGA1 expression may be a predictor of CRC tumorigenesis (Fig. S4G). Moreover, METTL16 positively regulated SOGA1 protein expression in vivo (Fig. 3R, S). Collectively, these results indicated that SOGA1 is a direct target of METTL16.

### IGF2BP1 is the m6A reader of SOGA1

**Fig. 4:**
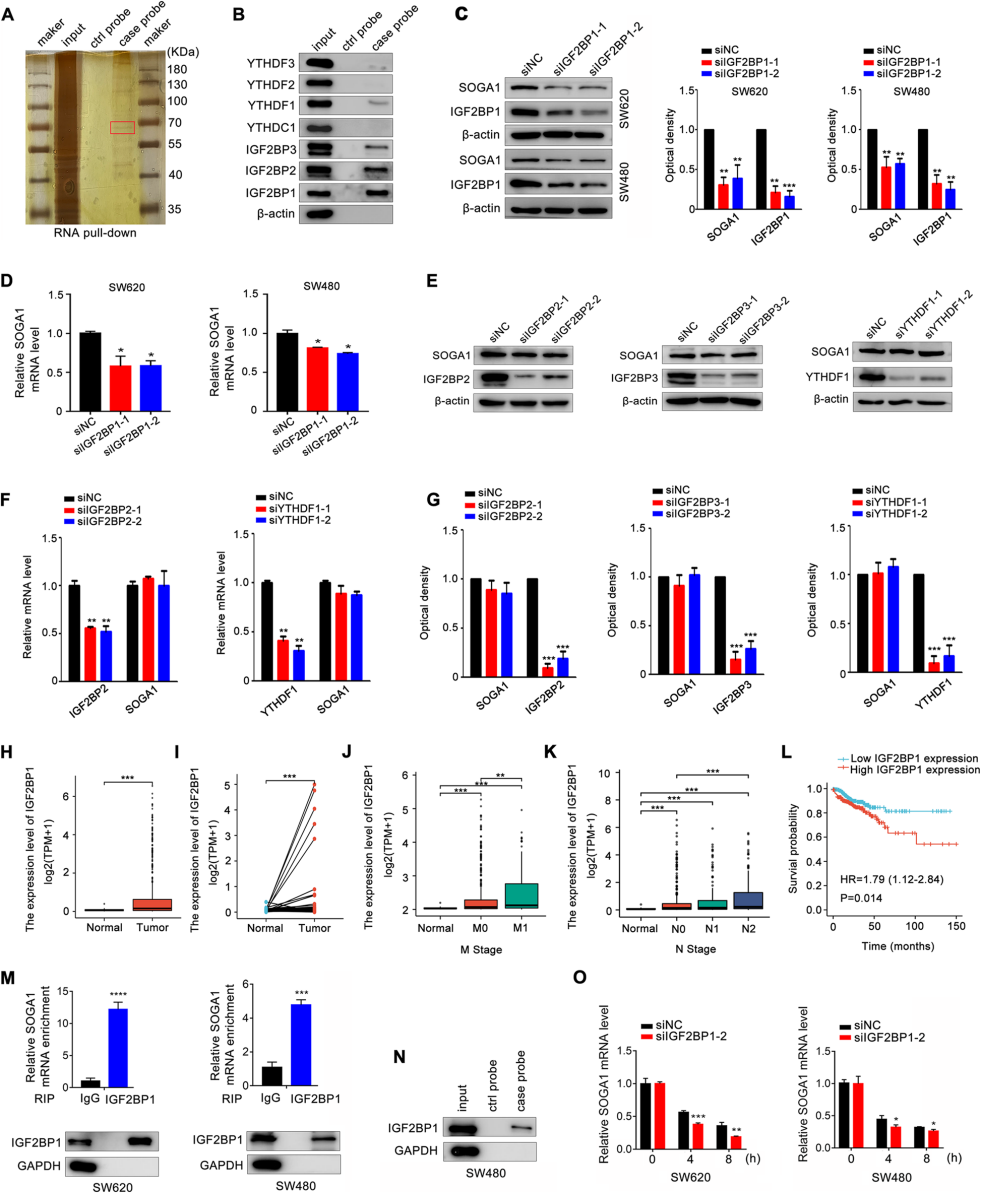
SOGA1 is specially recognized by IGF2BP1. **A** Silver staining revealed SOGA1-bound proteins from SW620 cells. **B** Immunoblotting of IGF2BP1/2/3, YTHDC1, YTHDF1/2/3 after RNA pull down assay with cell lysate (input), biotinylated-SOGA1 (case probe), and beads only (ctrl probe) in SW620 cells. **C** SOGA1 protein expression was detected in SW480 and SW620 cells with or without IGF2BP1 knockdown by western blotting. **D** SOGA1 mRNA expression was detected in SW480 and SW620 cells with or without IGF2BP1 knockdown by qRT-PCR. **E**, **G** SOGA1 protein expression in SW620 cells transfected with siRNAs of IGF2BP2, IGF2BP3 or YTHDF1 was examined by western blotting respectively. **F** SOGA1 mRNA expression was detected in SW620 cells with or without IGF2BP2 or YTHDF1 knockdown by qRT-PCR. (H, I) IGF2BP1 expression in the TCGA CRC cohort. **J**, **K** Association of IGF2BP1 mRNA expression with lymph node metastasis (**J**), and distant metastasis (**K**) in CRC patients in TCGA database. **L** Kaplan-Meier survival curves of OS based on IGF2BP1 expression in CRC patients in TCGA database. **M** RIP-qPCR displayed the relative enrichment of SOGA1 mRNA in each group precipitated with lgG or IGF2BP1 antibody with the normalization to input. IP efficiency of IGF2BP1 was validated using western blotting. GAPDH was used as protein control. **N** The capacity of IGF2BP1 binding to SOGA1 in SW480 cells was detected by RNA pulldown assay. **O** Stability of SOGA1 mRNA was detected in IGF2BP1-konckdown and control cells via qRT-PCR at the indicated time after actinomycin D (5 µg/ml) treatment. *P < 0.05, **P < 0.01, ***P < 0.001, ****P < 0.0001

The mechanisms responsible for m6A modified SOGA1 mRNA were investigated. It has been shown that m6A readers play an irreplaceable role in regulating mRNA modifications [25]. To identify the m6A reader that recognizes and binds SOGA1 methylation, an RNA pull-down assay was conducted to capture SOGA1-interacting readers from the SW620 cells. The m6A reader YTHDF1 and IGF2BP1/2/3, but not the others, were found to combine with SOGA1 mRNA (Fig. 4A, B). Interestingly, knockdown of IGF2BP1 significantly downregulated SOGA1 mRNA and protein levels in CRC cells (Fig. 4C, D). However, knockdown of YTHDF1 and IGF2BP2/3 had no obvious effect on SOGA1 protein expression (Fig. 4E-G and Fig. S5B). Analysis TCGA data revealed that IGF2BP1 expression was upregulated in CRC tissues compared with normal tissues (Fig. 4H, I). Increased expression of IGF2BP1 correlated significantly with the tumor size, lymph node metastasis, distant metastasis, and clinical stage grade (Fig. 4J, K and Fig. S5C, D). The ROC curve showed that IGF2BP1 expression may be a predictor of CRC tumorigenesis (Fig. S5E) and increased IGF2BP1 expression is associated with poor survival in CRC patients (Fig. 4L). In addition, IGF2BP1 mRNA expression was positively correlated with SOGA1 mRNA in CRC (Fig. S5F). Moreover, an RNA pulldown assay revealed a close interaction between IGF2BP1 protein and SOGA1 mRNA (Fig. 4M). Consistently, the results of the RIP assay further confirmed that IGF2BP1 directly binds to SOGA1 mRNA (Fig. 4N). As IGF2BP1 has been recognized to mainly regulate mRNA stability as an m6A reader, the effect of IGF2BP1 deficiency on the decay rate of SOGA1 mRNA was assessed. As shown in Fig. 4O, IGF2BP1 knockdown decreased the stability of SOGA1 mRNA and elevated its degradation rate in CRC cells. Taken together, these results indicate that the methylated SOGA1 mRNA is directly recognized by IGF2BP1, which inhibits transcript degradation and promotes SOGA1 expression in an m6A dependent manner.

### METTL16/SOGA1 promotes glycolysis via regulating pyruvate dehydrogenase kinase 4 (PDK4) expression

**Fig. 5:**
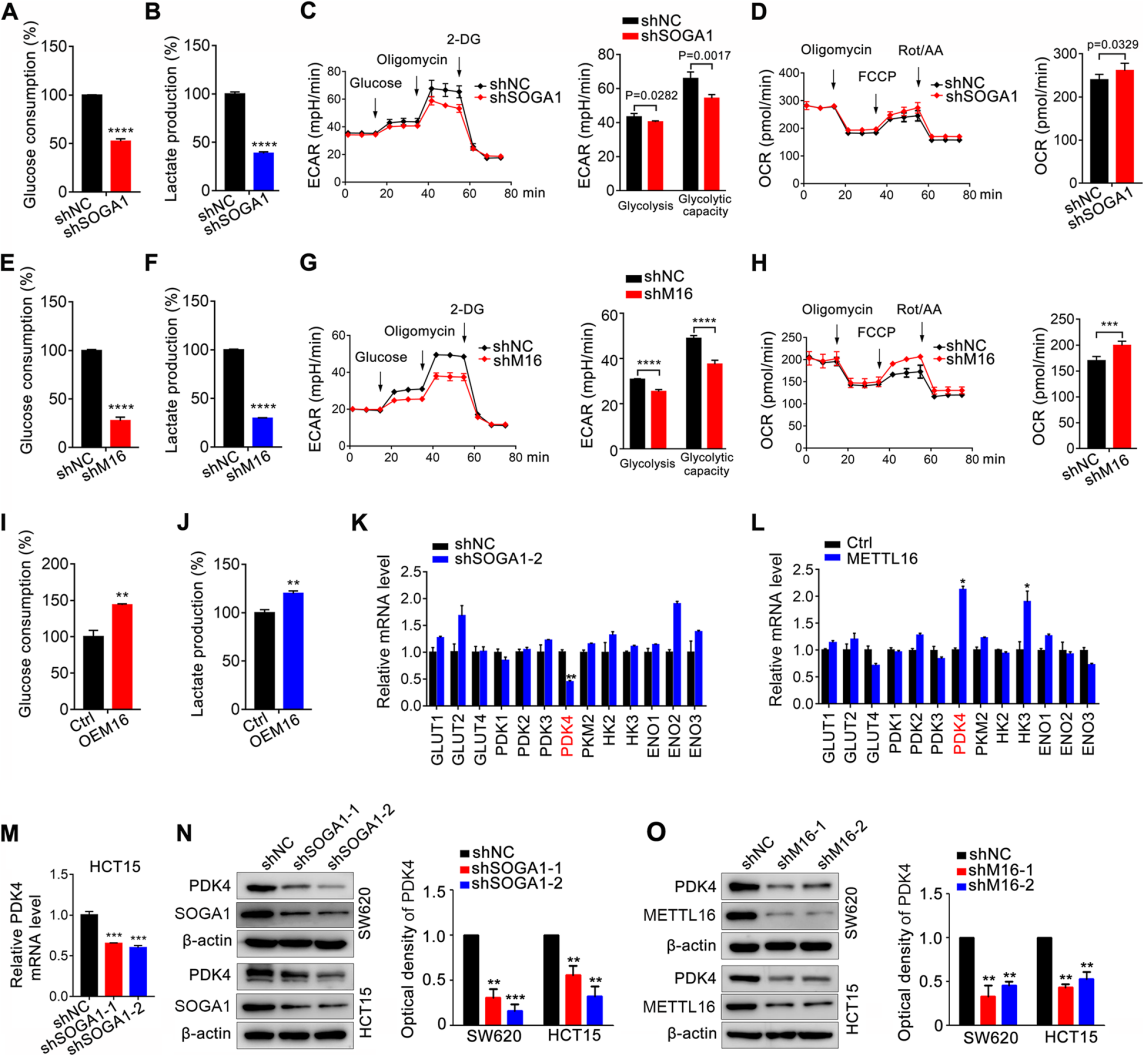
METTL16/SOGA1 axis enhances glycolysis by targeting PDK4 in CRC cells. **A**-**B** SOGA1 knockdown decreased glucose consumption (**A**) and lactate production (**B**) in SW620 cells. **C**-**D** The ECAR (**C**) and OCR (**D**) profile were measured in SOGA1 knockdown SW620 cells with a Seahorse XF24 analyser. The metabolic inhibitors were injected sequentially at different time points as indicated. **E**-**F** METTL16 knockdown decreased glucose consumption (**E**) and lactate production **F** in SW620 cells. **G**-**H** The ECAR (**G**) and OCR (**H**) profile were measured in METTL16 knockdown SW620 cells. **I**-**J** METTL16 overexpression increased glucose consumption (**I**) and lactate production (**J**) in SW620 cells. **K**-**L** PDK4 was identified as METTL16/SOGA1 regulated gene. The expression of a panel of glucose metabolism-related genes was detected by qRT-PCR in SOGA1 knockdown (**K**) or METTL16 overexpression (**L**) cells and their corresponding control cells. **M** PDK4 mRNA expression in HCT15 cells with SOGA1 knockdown was detected by qRT-PCR. **N** PDK4 protein expression in HCT15 and SW620 cells with SOGA1 knockdown was detected by western blotting. **O** PDK4 protein expression in HCT15 and SW620 cells with METTL16 knockdown was detected by western blotting. **P < 0.01, ***P < 0.001, ****P < 0.0001

SOGA1 is involved in glucose metabolism by inhibiting autophagy in hepatocytes [26]. Glycolysis is one of the most important processes in glucose metabolism in cancer ^3^. Therefore, the effect of METTL16/SOGA1 axis on glycolysis to promote CRC progression was investigated. Knockdown of SOGA1 was first confirmed (Fig. S6A), and knockdown of SOGA1 in SW620 cells markedly reduced glucose uptake and lactate production (Fig. 5A, B). Further, SOGA1 loss CRC cells showed decreased ECAR, an indicator of overall glycolytic flux, and increased OCR, reflecting mitochondrial oxidative respiration (Fig. 5C, D). Consistently, METTL16 deficiency CRC cells displayed decreased glucose uptake (Fig. 5E), lactate production (Fig. 5F), and ECAR (Fig. 5G), and increased OCR (Fig. 5H). In contrast, overexpression of METTL16 in SW620 cells promoted glucose uptake and lactate production (Fig. 5I, J). To further determine the mechanism underlying METTL16/SOGA1 mediated glycolysis, the mRNA expression of a series of glucose metabolism-related genes in SOGA1 knockdown CRC cells were measured (Fig. 5K). We also investigated whether these molecules were controlled by METTL16, so we detected these genes mRNA expression in METTL16 overexpressing-CRC cells (Fig. 5L). Intriguingly, the expression level of only PDK4 was dramatically decreased by SOGA1 knockdown and was consistently increased by METTL16 overexpression. Through further verification, we found that knockdown of both SOGA1 and METTL16 reduced PDK4 mRNA and protein expression in CRC cells (Fig. 5M-O and Fig. S6B), whereas overexpression of METTL16 significantly upregulated PDK4 expression in CRC cells (Fig. S6C). PDK controls the activity of pyruvate dehydrogenase by inhibitory phosphorylation on multiple residues, thereby preventing entry of glycolytic products into the TCA cycle, in parallel leading to reciprocal increased fatty acid oxidation [27]. As the most widely distributed PDKs isoform, PDK4 has been suggested as one of the most important factors controlling glucose metabolism via directing carbon flux into glycolysis from oxidative phosphorylation (OXPHOS) [28]. Several important studies have demonstrated the crucial role of PDK4 in glucose metabolism [29, 30]. Collectively, our data revealed that the METTL16/SOGA1 axis promoted glycolysis by regulating PDK4 expression in CRC cells.

### SOGA1 promotes PDK4 expression by suppressing AMP-activated protein kinase (AMPK) signaling

**Fig. 6:**
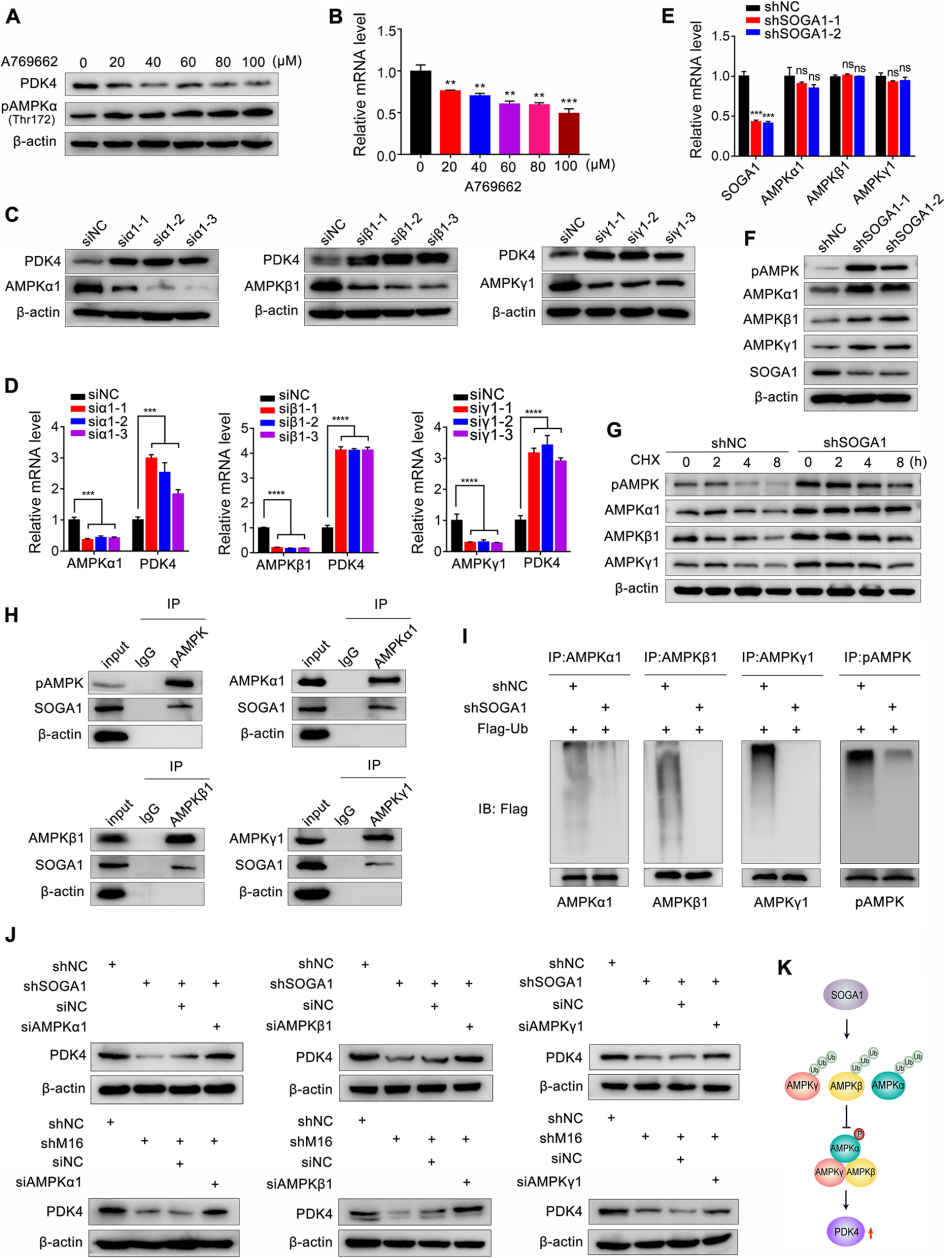
SOGA1 regulates PDK4 expression via inhibiting AMPK activation. **A**-**B** The PDK4 protein (**A**) and mRNA (**B**) expression in SW620 cells treated with different concentration of AMPK activator A769662 were measured by western blotting and qRT-PCR, respectively. **C**-**D** The PDK4 protein (**C**) and mRNA (**D**) expression in SW620 cells with or without AMPKα1, β1, γ1 knockdown (siα1, siβ1, siγ1) were measured by western blotting and qRT-PCR, respectively. **E**-**F** AMPKα1, β1, γ1 mRNA (**E**) and protein (**F**) expression in SW620 cells with or without SOGA1 knockdown were measured by qRT-PCR and western blotting, respectively. **G** The protein stability of pAMPK, AMPKα1, β1, γ1 were detected in SOGA1-konckdown and control cells via western blotting at the indicated time after CHX (100 µg/ml) treatment. **H** The capacity of SOGA1 binding to pAMPK, AMPKα1, β1, γ1 were detected by Co-IP. **I** The ubiquitylation of AMPKα1, β1, γ1, pAMPK were detected in SOGA1-konckdown and control cells by Co-IP. **J** The PDK4 protein expressions in SOGA1 or METTL16 deficient SW620 cell transfected with siRNAs of AMPKα1, β1, γ1 respectively were detected by western blotting. **K** A working model of SOGA1 regulating PDK4 expression. **P < 0.01, ***P < 0.001, ****P < 0.0001

We further explored the underlying mechanisms involved in SOGA1-regulated PDK4 expression. It has been reported that AMPK signaling is the key upstream of PDK4 [31]. To verify the role of AMPK in PDK4 expression, an AMPK activator A769662 was used to stimulate CRC cells. The results showed that A769662 induced AMPK (Thr172) phosphorylation, activated AMPK signaling and observably reduced PDK4 protein and mRNA expression (Fig. 6A, B and Fig. S7A). AMPK is a tri-complex consisting of an α catalytic subunit and β and γ regulatory subunits [32]. We found that knockdown of AMPKα1, β1 and γ1 subunits increased PDK4 mRNA and protein expression (Fig. 6C, D and Fig. S7B), suggesting that AMPK is upstream of PDK4 and negatively regulates PDK4 expression in CRC cells. Next, we found that knockdown of SOGA1 significantly promoted AMPK phosphorylation and AMPKα1, β1, and γ1 protein expression (Fig. 6F and Fig. S7C), but had no obvious effect on AMPKα1, β1 and γ1 mRNA expression (Fig. 6E), indicating that SOGA1 might regulate AMPK protein stability. The protein stability assay showed that SOGA1 knockdown reduced the decay of pAMPK, AMPKα1, β1 and γ1 and enhanced their protein stability (Fig. 6G and Fig. S7D). Previous studies have shown that AMPK is activated by various kinase, such as LKB1, CaMKK2, and TAK1 [32]. However, knockdown of LKB1, CaMKK2, and TAK1 had no obvious effect on SOGA1 loss mediated-AMPK phosphorylation (Fig. S7E-H), suggesting that there exists the other regulatory mechanism underlying SOGA1 mediated-AMPK activation. A recent study revealed that Circ-ACC1 can directly combine with AMPKβ and γ subunits, thus promoting AMPK phosphorylation and protein stability [32]. Similarly, Co-IP assay results showed that SOGA1 could bind to pAMPK, AMPKα1, β1, γ1 (Fig. 6H) and promote AMPK α1, β1, γ1, and pAMPK ubiquitination (Fig. 6I). Moreover, inhibition of AMPKα1, β1, and γ1 partly reversed SOGA1 or METTL16 loss downregulated-PDK4 expression (Fig. 6J Fig. S7I). Taken together, these results demonstrated that SOGA1 bind to AMPKα1, β1, and γ1, inducing their ubiquitination and inhibiting their expression and phosphorylation, thereby promoting PDK4 expression (Fig. 6K).

### Yin Yang 1 (YY1) downregulates METTL16 expression by directly binding to its promoter

**Fig. 7:**
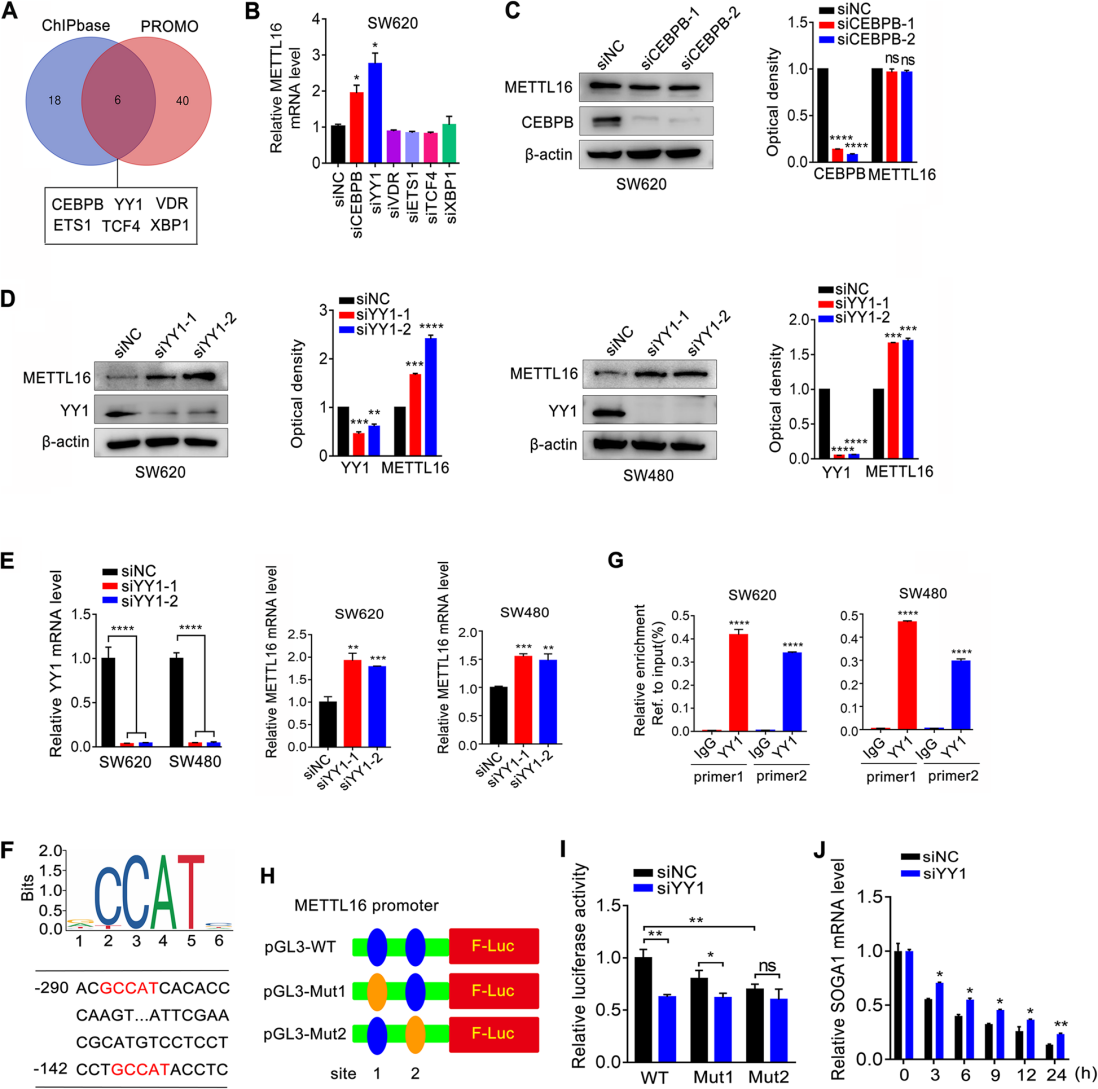
YY1 transcriptionally regulates METTL16 expression in CRC cells. **A** Venn diagram showed the possible transcription factors of METTL16 predicted by PROMO and ChIPBase. **B** Relative mRNA expression of METTL16 in SW620 cells with CEBPB, YY1, VDR, ETS1, TCF4 or XBP1 knockdown was measured by qRT-PCR. **C** METTL16 protein expression in SW620 cells with CEBPB knockdown was measured by western blotting. **D** METTL16 protein expression in SW620 and SW480 cells with YY1 knockdown was measured by western blotting. **E** METTL16 mRNA expression in SW620 and SW480 cells with YY1 knockdown was measured by qRT-PCR. **F** The YY1 binding sites in METTL16 promoter. (**G**) ChIP assay was performed in SW620 and SW480 cells to detect the ability of YY1 binding to the METTL16 promoter. **H** Schematic representation of the mutated promoter in pGL3-Basic-METTL16-luc reporter to investigate the role of YY1 in METTL16 expression. **I** SW620 cells were co-transfected with pGL3-METTL16-WT-Luc, pGL3-METTL16-Mut1-Luc, pGL3-METTL16-Mut2-Luc, pRL-TK and si-NC or si-YY1 for 24 h. Results were presented as the ratio between the activity of the reporter plasmid and pRL-TK. **J** Stability of SOGA1 mRNA was detected in SW620 cells with YY1-konckdown and control cells via qRT-PCR at the indicated time after actinomycin D (5 µg/ml) treatment. *P < 0.05, **P < 0.01, ***P < 0.001, ****P < 0.0001

To explore the underlying mechanism of high METTL16 expression in CRC, we evaluated the potential transcription factors (TFs) responsible for the regulation of METTL16 by analyzing ENCODE chromatin immunoprecipitation sequencing (ChIP-seq) data in ChIPBase and PROMO. As shown in Fig. 7A, six TFs, CEBPB, YY1, VDR, ETS1, TCF4, and XBP1 overlapped in 26 TFs predicted by ChIPBase and 46 TFs predicted by PROMO. Next, these six TFs were downregulated respectively, and found that CEBPB and YY1 knockdown, but not the others, increased METTL16 mRNA expression (Fig. 7B). However, loss of CEBPB had no obvious effect on METTL16 protein expression (Fig. 7C). YY1 knockdown obviously upregulated METTL16 protein and mRNA expression in CRC cells (Fig. 7D, E). In an analysis of the METTL16 gene promoter, we found the YY1 binding sites on it and designed ChIP primers (Fig. 7F). The ChIP assay showed that YY1 could directly bind to the METTL16 promoter in CRC cells (Fig. 7G), indicating that YY1 was the upstream TF of METTL16. Next, we mutated the two YY1 potential binding sites of promoter reporter of METTL16 to generate the pGL-M16-Mut1 or pGL-M16-Mut2 (Fig. 7H). The results showed that si-YY1 can significantly decrease luciferase levels of pGL-M16-WT and pGL-M16-Mut1, while the inhibition effect of si-YY1 was attenuated for pGL-M16-Mut2 (Fig. 7I). Moreover, YY1 knockdown increased the SOGA1 mRNA stability in CRC cells (Fig. 7J). Taken together, these data demonstrated that YY1 is the upstream transcriptional factor of METTL16 and regulates METTL16 expression by directly binding to its promoter.

### The METTL16/SOGA1 axis are clinically relevant with poor prognosis in CRC patients

**Fig. 8:**
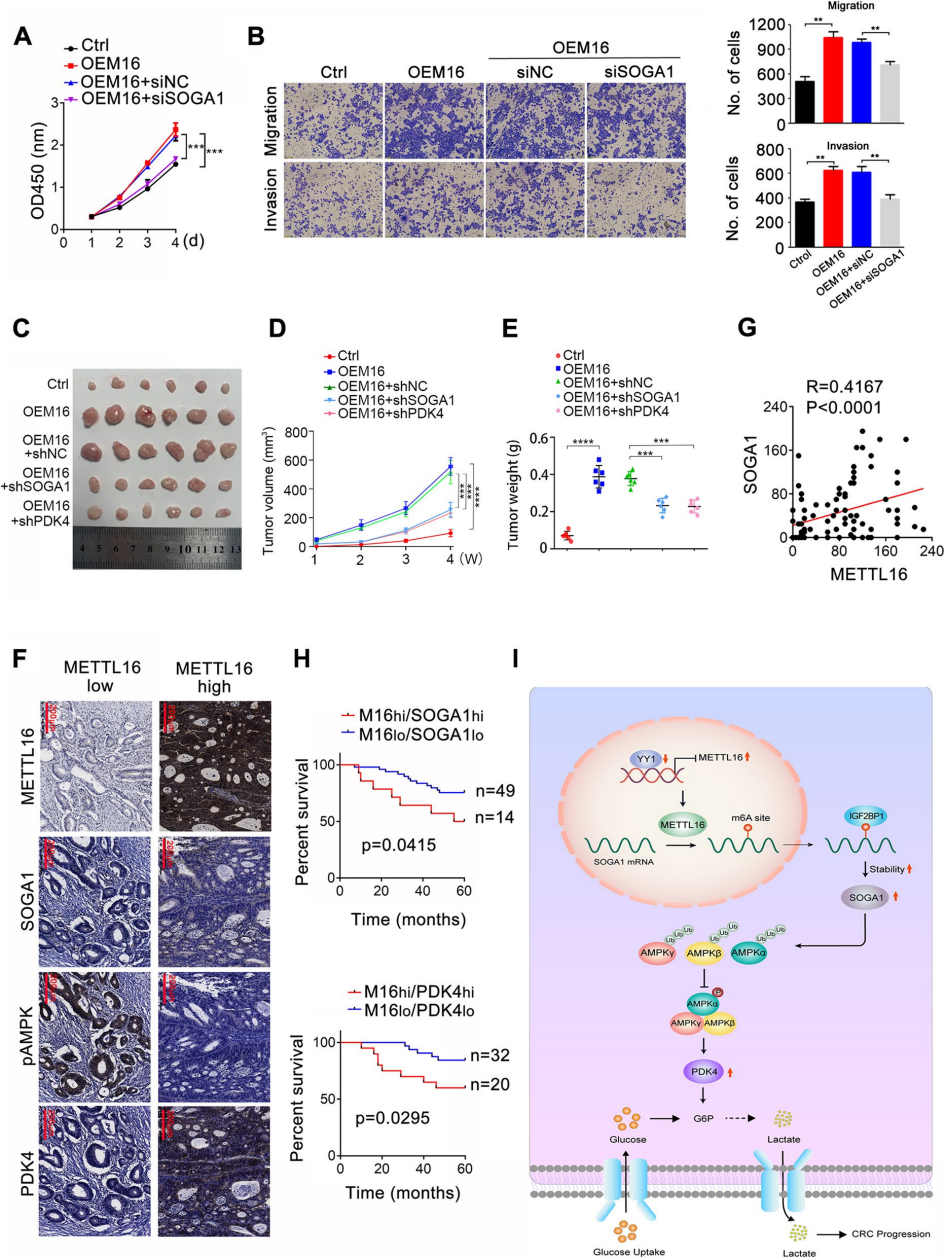
METTL16/SOGA1 axis are clinically relevant with poor prognosis in CRC patients. **A** CCK8 assay was conducted to measure proliferation of SW620-OEM16 cells transfected with siSOGA1. **B** Transwell assays were conducted to measure migration and invasion of SW620-OEM16 cells transfected with siSOGA1. **C** Xenografts derived from SW620-OEM16 or SW620-OEM16-shSOGA1/shPDK4 cells and relative controls (*n* = 6). **D**, **E** Tumor volume was recorded at indicated time to establish a growth curve (**D**) and tumors weight (**E**) were measured after mice sacrificed. (**F**) Representative images of METTL16, SOGA1, pAMPK, and PDK4 IHC staining in CRC tissues. **G** Correlation analysis between METTL16 and SOGA1 protein expression based on IHC staining scores. **H** Kaplan-Meier survival curves of OS based on METTL16/SOGA1 or METTL16/PDK4 IHC staining in CRC patients. **I** A working model of METTL16-promoted SOGA1 m6A modification in CRC progression. **P < 0.01, ***P < 0.001

To investigate the role of SOGA1 in METTL16 mediated-CRC proliferation, we downregulated SOGA1 expression using siRNA in METTL16 overexpressing CRC cells and examined cell proliferation. The results showed that SOGA1 knockdown weakened METTL16 promoted-CRC cells proliferation (Fig. 8A). Knockdown of AMPKα1, β1, and γ1, and overexpression of SOGA1 displayed a similar function, partly reversed METTL16 mediated-CRC proliferation (Fig. S8A). Consistently, SOGA1 knockdown partly inhibited METTL16 promoted-CRC cells metastasis (Fig. 8B). In vivo, SOGA1 and PDK4 knockdown both weakened overexpression of METTL116 elevated tumor growth, as reflected by tumor size (Fig. 8C), volume (Fig. 8D), and weight (Fig. 8E). Moreover, the expression of METTL16 and SOGA1 was positively correlated in CRC tissues (Fig. 8F, G and Fig. S8B). TCGA database also showed a significant positive correlation between METTL16 and SOGA1 mRNA expression (Fig. S8B). In addition, METTL16 expression was positively correlated with PDK4 in CRC tissues (Fig. 8F and Fig. S8B). Importantly, Kaplan–Meier analysis showed that co-expression of METTL16 and SOGA1 or METTL16 and PDK4 at high expression levels positively correlated with poor prognosis in patients with CRC (Fig. 8H). The above results suggest that SOGA1 play an important role in proliferation mediated by METTL16 in CRC cells and that the METTL16/SOGA1 axis is clinically relevant with poor prognosis in CRC patients.

## Discussion

To date, more than 100 types of post-transcriptional modifications of human RNA have been identified [33, 34]. As the most abundant RNA modification, m6A methylation mediated-RNA metabolism is of great significance for mRNA and non-coding RNA expression regulation and has become a critical research hotspot in recent years [35, 36]. Methyltransferases, demethylases, and RNA binding proteins control m6A modification and regulate RNA splicing, translation, export, decay, and stability [37]. Several studies have shown that m6A modification plays an essential role in the initiation and progression of various cancers [38, 39, 40]. In the present study, we found that m6A methyltransferase METTL16 expression was remarkably elevated in CRC and correlated with poor prognosis. METTL16 promoted CRC proliferation in vitro and in vivo and induced glycolysis in CRC cells. Mechanistically, the transcription factor YY1 directly binds to METTL16 promoter and inhibits its expression, which upregulates SOGA1 by promoting m6A modification of SOGA1 mRNA and enhancing its mRNA stability in an IGF2BP1 dependent manner. Increased SOGA1 further upregulated PDK4 by promoting AMPK ubiquitination and suppressing its phosphorylation, thereby mediated METTL16 induced CRC glycolysis and progression (Fig. 8I).

METTL16 is a novel m6A methyltransferase and its known m6A targets include U6 RNA, MALAT1, and MAT2A [41, 42, 43, 44, 45]. Although previous studies have shown the function of METTL16 in mouse embryonic development [46], the biological roles of METTL16 in diseases, especially tumor progression, are not well understood. A recent study has shown that METTL16 promotes gastric cancer proliferation by up-regulating cyclin D1 expression [47]. In addition, Su et al. reported that METTL16 display an m6A-independent function to promote transcript translation and contributed to hepatoma carcinoma cell tumorigenesis [48]. Herein, we found that METTL16 plays a crucial tumor-promoting role in CRC. Although the important regulatory functions of METTL3 and METTL14 in CRC development have been proven, mass-data analysis showed that METTL16 is most closely relevant to CRC progression among the METTL family molecules. METTL16 expression is significantly increased in CRC and associated with poor prognosis, suggesting that METTL16 may be a promising diagnosis biomarker for CRC. In vitro and in vivo experiments demonstrated that METTL16 promoted CRC tumor growth depending on its m6A catalytic activity. Furthermore, through database analysis and experimental validation, YY1 was identified as an upstream transcription factor of METTL16. Therefore, the YY1/METTL16 axis may serve as a potential therapeutic target for CRC treatment.

Using RNA-sequencing and MeRIP-sequencing, SOGA1 was identified as the pivotal target of METTL16 in CRC. METTL16 directly binds to SOGA1 mRNA, induces its m6A modification, and enhances mRNA stability, thus promoting SOGA1 protein expression. Moreover, RNA pulldown and RIP assays demonstrated that IGF2BP1, but not the other readers, could bind to SOGA1 mRNA and regulate its protein expression, suggesting that IGF2BP1 is a key reader mediating SOGA1 m6A modification in CRC. SOGA1 is known as an autophagy suppressor and is involved in the adiponectin-mediated inhibition of glucose production by inhibiting autophagy in an insulin-dependent manner in hepatocytes [26]. Kruse et al. found that SOGA1 is a microtubule associated protein that can bind glycogen synthase and glycogenin, an important biosynthetic enzyme for glycogen synthesis [49]. SOGA1 may modulate glucose and glycogen metabolism by directly cooperating with glycogen synthase and glycogenin [49]. In hepatoma carcinoma, SOGA1 mRNA is dramatically upregulated and may serve as a diagnostic and prognostic biomarker [50]. In this study, we showed that SOGA1 expression was obviously increased in CRC and inhibition of SOGA1 diminished CRC progression mediated by METTL16. Recently, increasing researches has proved the pivotal role of m6A modulators in the glucose metabolism of tumors [51]. For example, METTL3 promotes gastric cancer glycolysis and progression by regulating the m6A modification of hepatoma-derived growth factor (HDGF) mRNA [52]. METTL3 mediated-Hexokinase 2 (HK2) and glucose transporter type 1 (GLUT1) expression also induces CRC glucose metabolism [53]. Similarly, our study found that the METTL16/SOGA1 axis can accelerate glucose metabolism in CRC cells. Through screening a series of glucose metabolism-related genes, PDK4 was verified can be regulated by METTL16/SOGA1. PDK4 is recognized as one of the most crucial proteins regulating glucose metabolism by guiding carbon flux into glycolysis via oxidative phosphorylation [28]. It has been reported that PDK4 played oncogenic effects in CRC and bladder cancer [54, 55]. A recent study showed that METTL3 and ALKBH5 regulate mRNA stability and translation of PDK4, which mediates m6A induced-glycolysis and ATP production [29]. Here, we found that the METTL16/SOGA1 promotes glucose uptake and lactate generation in CRC cells and increases PDK4 expression. In addition, AMPK has been proved as the upstream regulator of PDK4 in muscle, cardiomyocytes, and decidual cells [31]. The role of AMPK in glucose metabolism and tumor progression has been widely confirmed [56, 57, 58]. In this study, we demonstrated that SOGA1 promotes ubiquitination of AMPK subunits α, β, γ and inhibits their expression, subsequently suppressing the AMPK holoenzyme phosphorylation and decreasing its enzymatic activity, thus inducing PDK4 expression in CRC cells. Clinically, METTL16 expression was positively correlated with SOGA1 and PDK4 expression in CRC tissues, suggesting the clinical significance of the METTL16/SOGA1/PDK4 axis in promoting CRC progression. Our study revealed a novel regulatory mechanism for METTL16 in CRC development.

## Conclusions

Our findings indicate a tumor-promoting role of METTL16 in CRC progression. METTL16 is upregulated in CRC tissues and is associated with poor prognosis in CRC patients. Mechanistically, the METTL16/SOGA1/PDK4 signaling axis promotes CRC progression by inducing glycolysis. This discovery provides new insights into the exploration of new diagnostic biomarkers and therapeutic targets for CRC.

### Supplementary Information

**Additional file 1:** **Figure S1.** (A) METTL16 expression in the GSE37182 CRC database. (B) Association of METTL16 mRNA expression with pathologic stage in CRC patients in TCGA database. (C) The ROC curve of METTL16 in predicting tumorigenesis of CRC. ****P<0.0001. **Figure S2.** (A-D) The knockdown and overexpression efficiency of METTL16 were detected by qRT-PCR and western blotting, respectively. (E-F) Transwell assays were performed to detect the migrative and invasive capacity of HCT15 cells with METTL16 knockdown (E) or overexpression (F). ***P<0.001, ****P<0.0001. **Figure S3.** (A) m6A peak number were detected in METTL16-knockdown group and control group. (B) Distribution and percentage of the m6A peaks of METTL16-knockdown group and control group in the genome. (C) Distribution and percentage of the differential peaks of METTL16-knockdown group and control group in the genome. (D) Metagene profiles of the differential m6A peaks. (E) The statistically upregulated (red) and downregulated (green) genes were exhibited via volcano plot. (F) M-A plot showed the upregulated genes (red) and downregulated genes (green) in RNA-sequencing data. (G) Function annotations of the differential mRNA in METTL16-knockdown group and control group by GO analysis. **Figure S4.** (A) SOGA1 mRNA expression in SW620, HCT116 and HCT15 cells treated with DAA was examined by qRT-PCR. (B-D) SOGA1 protein expression in SW620, HCT116 and HCT15 cells treated with DAA was examined by western blotting. (E) Immunoblotting analysis of SOGA1 expression in subcellular fractions of SW620 cells stable knockdown of METTL16 and control cells. (F) Association of SOGA1 mRNA expression with pathologic stage in CRC patients in TCGA database. (G) The ROC curve of SOGA1 in predicting tumorigenesis of CRC. *P<0.05, **P<0.01, ***P<0.001, ****P<0.0001. **Figure S5.** (A) The knockdown efficiency of IGF2BP1 was detected by qRT-PCR. (B) SOGA1 mRNA expression was detected in SW620 cells with or without IGF2BP3 knockdown by qRT-PCR. (C-D) Association of IGF2BP1 mRNA expression with tumor size (C) and pathologic stage (D) in CRC patients in TCGA database. (E) The ROC curve of IGF2BP1 in predicting tumorigenesis of CRC. (F) TCGA database showed the mRNA expression correlation between IGF2BP1 and SOGA1 in CRC tissues. ***P<0.001, ****P<0.0001. **Figure S6.** (A) The knockdown efficiency of SOGA1 in SW620 and HCT15 cells was detected by qRT-PCR. (B) PDK4 mRNA expression in SW620 and HCT15 cells with METTL16 knockdown were detected by qRT-PCR. (C) PDK4 protein expression in SW620 cells with METTL16 overexpression was detected by western blotting. **P<0.01, ***P<0.001, ****P<0.0001. **Figure S7.** (A) The quantitatively analysis of western blotting results about the PDK4 protein expression in SW620 cells treated with different concentration of AMPK activator A769662. (B) The quantitatively analysis of western blotting results about the PDK4 protein expression in SW620 cells with or without AMPKα1, β1, γ1 knockdown. (C) The quantitatively analysis of western blotting results about the AMPKα1, β1, γ1 protein expression in SW620 cells with or without SOGA1 knockdown. (D) The quantitatively analysis of western blotting results about the protein stability of pAMPK, AMPKα1, β1, γ1 in SOGA1-konckdown and control cells at the indicated time after CHX (100 μg/ml) treatment. (E) The knockdown efficiency of LKB1, CaMKK2, and TAK1 in SW620 cells was detected by qRT-PCR respectively. (F-H) The effects of inhibition of LKB1, CaMKK2, and TAK1 on pAMPK expression in SOGA1 deficient SW620 cells were detected by western blotting. (I) The quantitatively analysis of western blotting results about the PDK4 protein expressions in SOGA1 or METTL16 deficient SW620 cell transfected with siRNAs of AMPKα1, β1, γ1 respectively. **P<0.01, ***P<0.001, ****P<0.0001. **Figure S8.** (A) CCK8 assay was conducted to measure proliferation of SW620-shM16 cells transfected with siAMPKα1, β1, γ1, and SOGA1 overexpression vector, respectively. (B) Correlation analysis between the expression of METTL16 and SOGA1, METTL16 and PDK4, SOGA1 and PDK4, METTL16 and IGF2BP1 based on TCGA database.

**Additional file 2:** **Table S1.** The sequences of siRNAs or shRNAs.

**Additional file 3:** **Table S2.** Primers used in the study.

**Additional file 4:** **Table S3.** METTL16 expression in clinical and pathologicalcharacteristics of colorectal cancer patients.

**Additional file 5.**

## Data Availability

The authors declare that all the data supporting the findings in this study are available in this study and its Supplementary materials, or are available from the corresponding author through reasonable request.
