# Correction: METTL16 promotes glycolytic metabolism reprogramming and colorectal cancer progression

**DOI:** 10.1186/s13046-024-02951-x

**Published:** 2024-01-22

**Authors:** Wei Wei, Zhong-Yuan Zhang, Bin Shi, Yike Cai, Hou-Shun Zhang, Chun-Lei Sun, Yun-Fei Fei, Wen Zhong, Shuang Zhang, Chen Wang, Bing He, Guan-Min Jiang, Hao Wang

**Affiliations:** 1 Department of Laboratory Medicine, The First Affiliated Hospital of USTC, Division of Life Sciences and Medicine, University of Science and Technology of China, Hefei, China; 2 Core Unit of National Clinical Research Center for Laboratory Medicine, Hefei, China; 3 Department of Radiology, The First Affiliated Hospital of USTC, Division of Life Sciences and Medicine, University of Science and Technology of China, Hefei, China; 4 Department of General Surgery, The First Affiliated Hospital of USTC, Division of Life Sciences and Medicine, University of Science and Technology of China, Hefei, China; 5 Center for Certification and Evaluation, Guangdong Drug Administration, Guangzhou, China; 6 Department of Pathology, The First Affiliated Hospital of USTC, Division of Life Sciences and Medicine, University of Science and Technology of China, Hefei, China; 7 Department of Clinical Laboratory, The Fifth Affiliated Hospital, Sun Yat-sen University, Zhuhai, China

**Correction:** ***J Exp Clin Cancer Res*** **42, 151 (2023)**

10.1186/s13046-023-02732-y

Following publication of the original article [1], the authors would spotted errors in the affiliations. ‘The First Affiliated Hospital of USTC’ should come before the division. Affiliations 1, 3, 4, and 6 were corrected accordingly.

The corrections do not affect the overall result or conclusion of the article. The original article has been corrected.
