# Supplementary material for: METTL16 promotes glycolytic metabolism reprogramming and colorectal cancer progression
Source: J Exp Clin Cancer Res. 2023 Jun 20;42:151. doi: 10.1186/s13046-023-02732-y (PMC10280857; doi:10.1186/s13046-023-02732-y)
Supplement: Supplementary file 1 — Additional file 1: Figure S1. (A) METTL16 expression in the GSE37182 CRC database. (B) Association of METTL16 mRNA expression with pathologic stage in CRC patients in TCGA database. (C) The ROC curve of METTL16 in predicting tumorigenesis of CRC. ****P<0.0001. Figure S2. (A-D) The knockdown and overexpression efficiency of METTL16 were detected by qRT-PCR and western blotting, respectively. (E-F) Transwell assays were performed to detect the migrative and invasive capacity of HCT15 cells with METTL16 knockdown (E) or overexpression (F). ***P<0.001, ****P<0.0001. Figure S3. (A) m6A peak number were detected in METTL16-knockdown group and control group. (B) Distribution and percentage of the m6A peaks of METTL16-knockdown group and control group in the genome. (C) Distribution and percentage of the differential peaks of METTL16-knockdown group and control group in the genome. (D) Metagene profiles of the differential m6A peaks. (E) The statistically upregulated (red) and downregulated (green) genes were exhibited via volcano plot. (F) M-A plot showed the upregulated genes (red) and downregulated genes (green) in RNA-sequencing data. (G) Function annotations of the differential mRNA in METTL16-knockdown group and control group by GO analysis. Figure S4. (A) SOGA1 mRNA expression in SW620, HCT116 and HCT15 cells treated with DAA was examined by qRT-PCR. (B-D) SOGA1 protein expression in SW620, HCT116 and HCT15 cells treated with DAA was examined by western blotting. (E) Immunoblotting analysis of SOGA1 expression in subcellular fractions of SW620 cells stable knockdown of METTL16 and control cells. (F) Association of SOGA1 mRNA expression with pathologic stage in CRC patients in TCGA database. (G) The ROC curve of SOGA1 in predicting tumorigenesis of CRC. *P<0.05, **P<0.01, ***P<0.001, ****P<0.0001. Figure S5. (A) The knockdown efficiency of IGF2BP1 was detected by qRT-PCR. (B) SOGA1 mRNA expression was detected in SW620 cells with or without IGF2BP3 knockdown by qRT-PC [file 13046_2023_2732_MOESM1_ESM.doc]

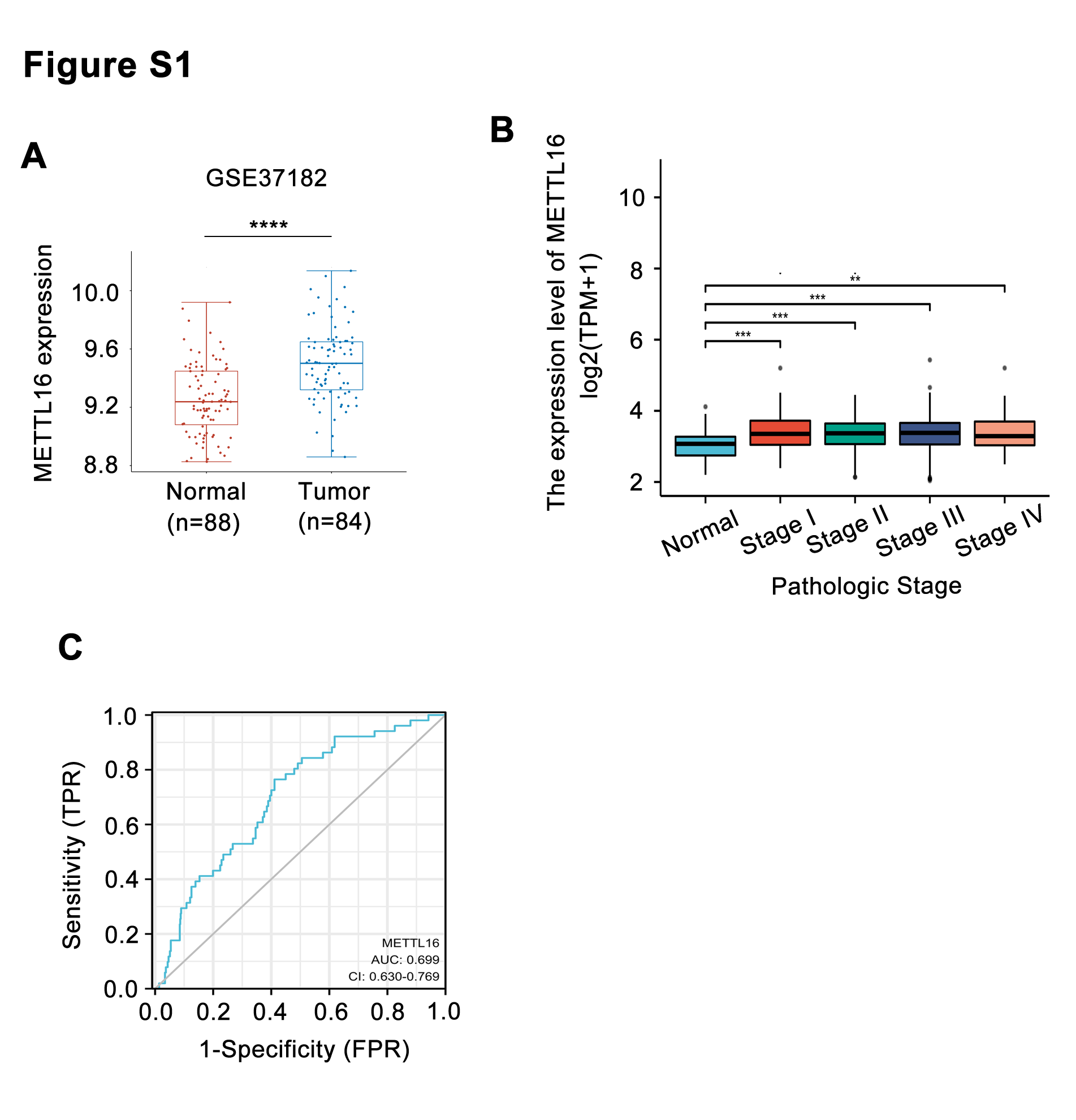


**Figure S1.** (A) METTL16 expression in the GSE37182 CRC database. (B) Association of METTL16 mRNA expression with pathologic stage in CRC patients in TCGA database. (C) The ROC curve of METTL16 in predicting tumorigenesis of CRC. ****P<0.0001.


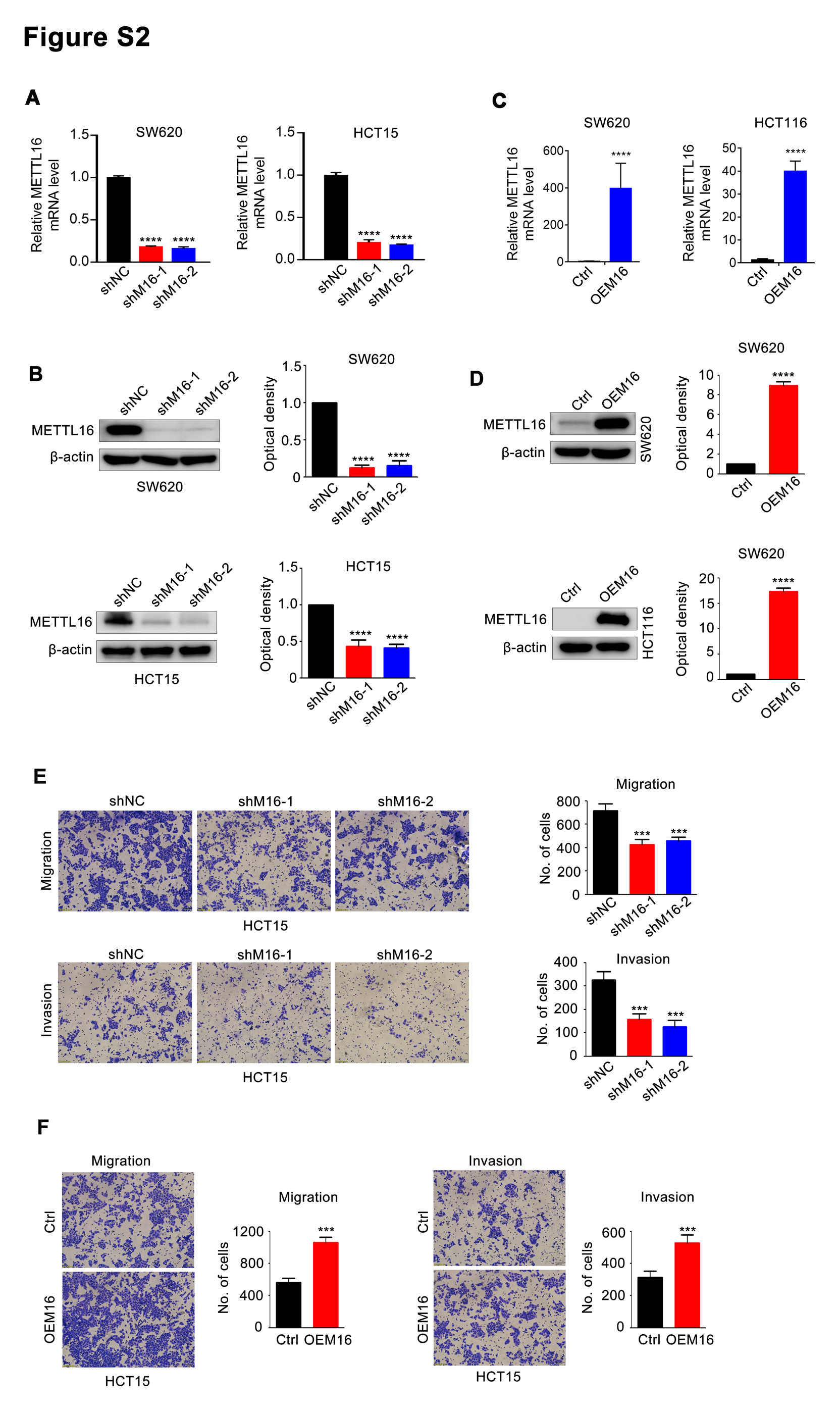


**Figure S2.** (A-D) The knockdown and overexpression efficiency of METTL16 were detected by qRT-PCR and western blotting, respectively. (E-F) Transwell assays were performed to detect the migrative and invasive capacity of HCT15 cells with METTL16 knockdown (E) or overexpression (F). ***P<0.001, ****P<0.0001.


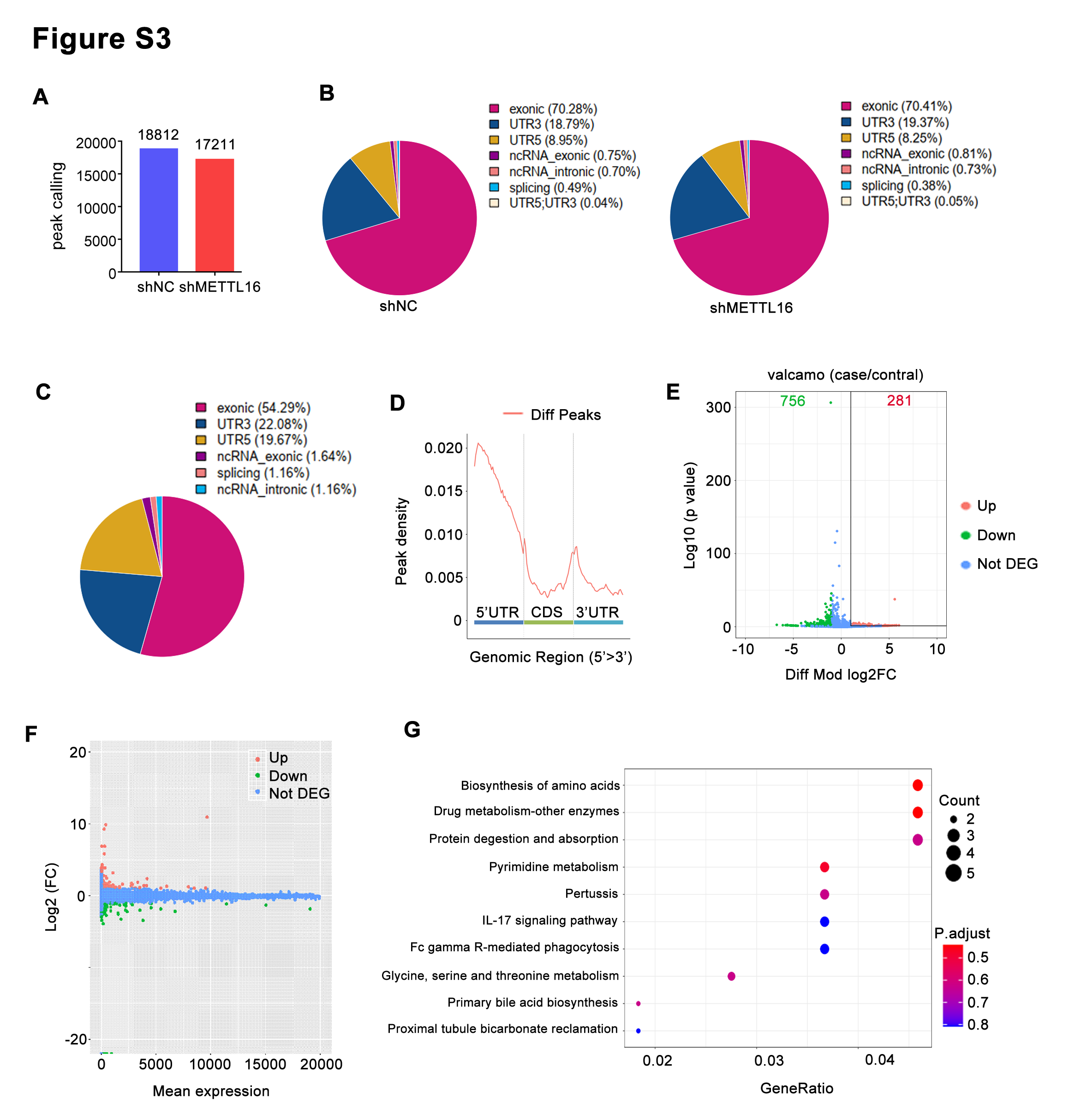


**Figure S3**. (A) m6A peak number were detected in METTL16-knockdown group and control group. (B) Distribution and percentage of the m6A peaks of METTL16-knockdown group and control group in the genome. (C) Distribution and percentage of the differential peaks of METTL16-knockdown group and control group in the genome. (D) Metagene profiles of the differential m6A peaks. (E) The statistically upregulated (red) and downregulated (green) genes were exhibited via volcano plot. (F) M-A plot showed the upregulated genes (red) and downregulated genes (green) in RNA-sequencing data. (G) Function annotations of the differential mRNA in METTL16-knockdown group and control group by GO analysis.


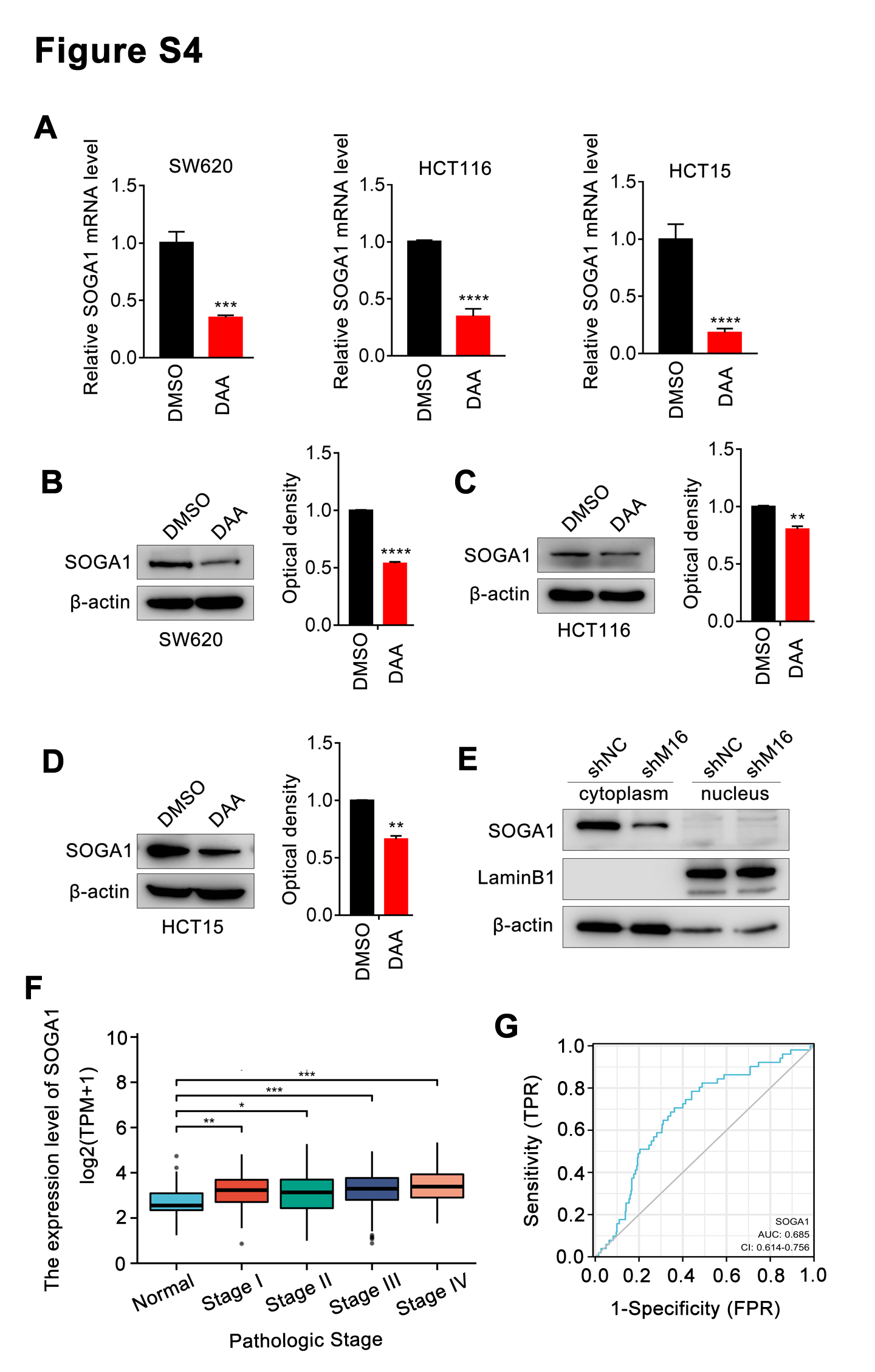


**Figure S4.** (A) SOGA1 mRNA expression in SW620, HCT116 and HCT15 cells treated with DAA was examined by qRT-PCR. (B-D) SOGA1 protein expression in SW620, HCT116 and HCT15 cells treated with DAA was examined by western blotting. (E) Immunoblotting analysis of SOGA1 expression in subcellular fractions of SW620 cells stable knockdown of METTL16 and control cells. (F) Association of SOGA1 mRNA expression with pathologic stage in CRC patients in TCGA database. (G) The ROC curve of SOGA1 in predicting tumorigenesis of CRC. *P<0.05, **P<0.01, ***P<0.001, ****P<0.0001.


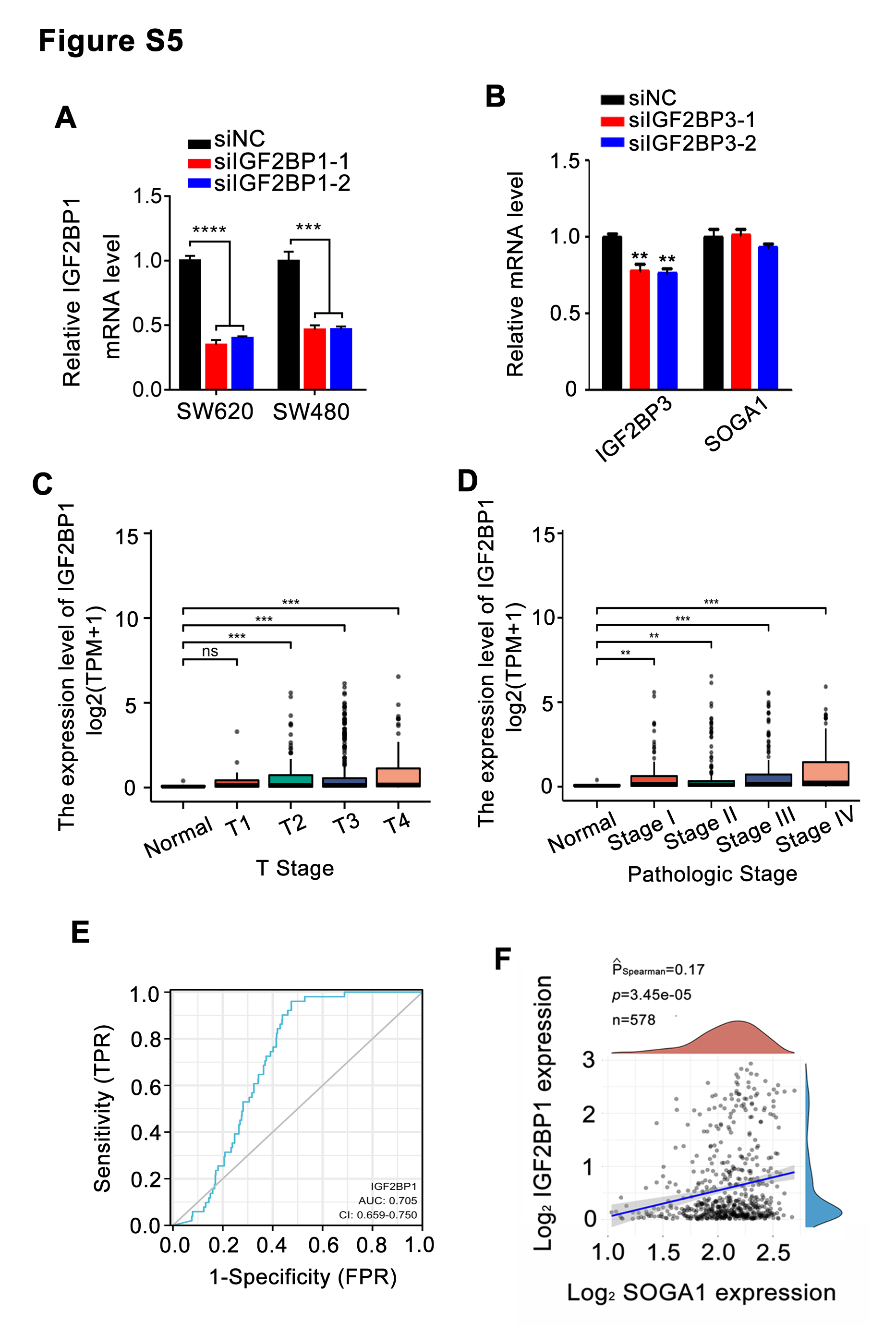


**Figure S5**. (A) The knockdown efficiency of IGF2BP1 was detected by qRT-PCR. (B) SOGA1 mRNA expression was detected in SW620 cells with or without IGF2BP3 knockdown by qRT-PCR. (C-D) Association of IGF2BP1 mRNA expression with tumor size (C) and pathologic stage (D) in CRC patients in TCGA database. (E) The ROC curve of IGF2BP1 in predicting tumorigenesis of CRC. (F) TCGA database showed the mRNA expression correlation between IGF2BP1 and SOGA1 in CRC tissues. ***P<0.001, ****P<0.0001.


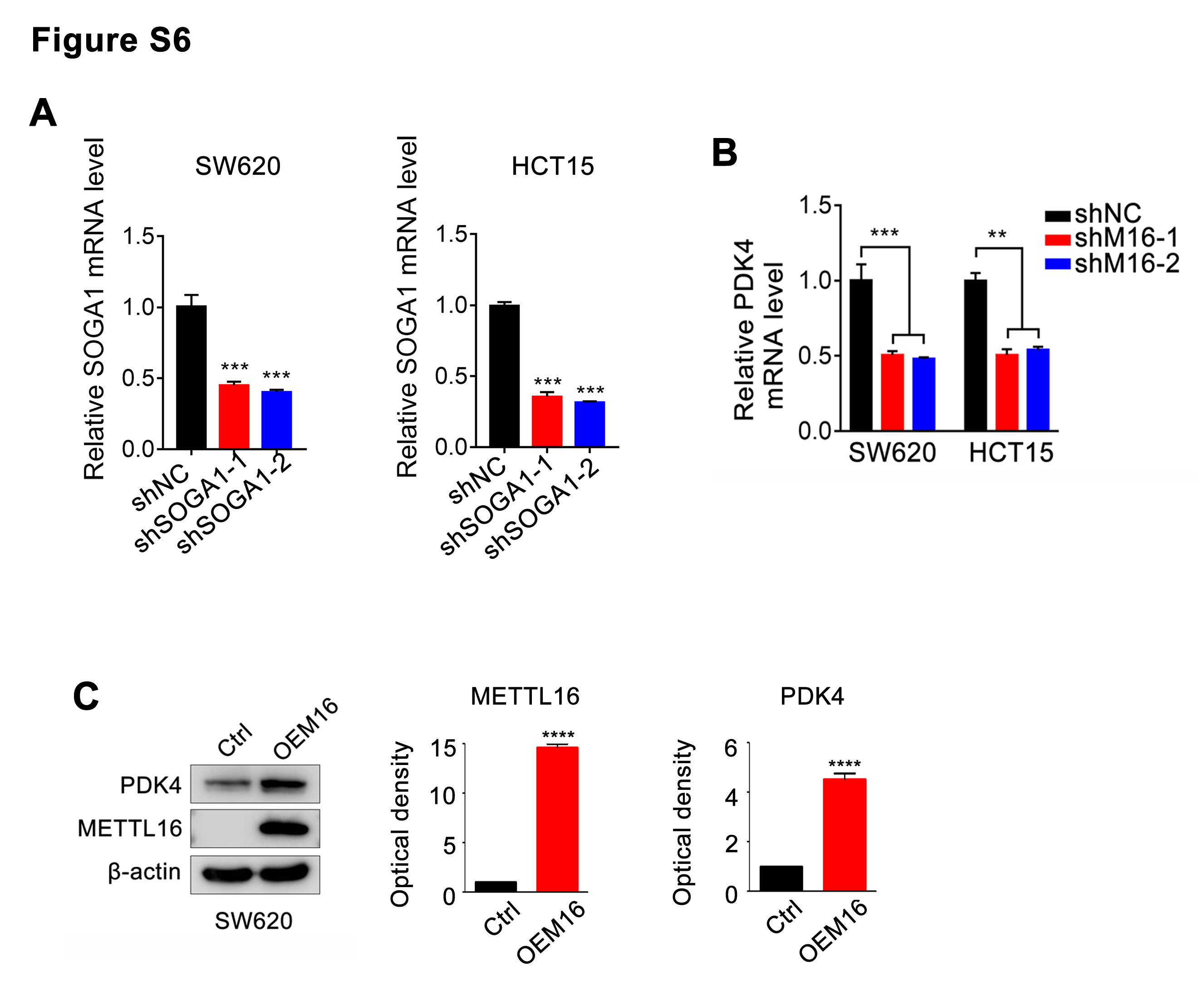


**Figure S6**. (A) The knockdown efficiency of SOGA1 in SW620 and HCT15 cells was detected by qRT-PCR. (B) PDK4 mRNA expression in SW620 and HCT15 cells with METTL16 knockdown were detected by qRT-PCR. (C) PDK4 protein expression in SW620 cells with METTL16 overexpression was detected by western blotting. **P<0.01, ***P<0.001, ****P<0.0001.


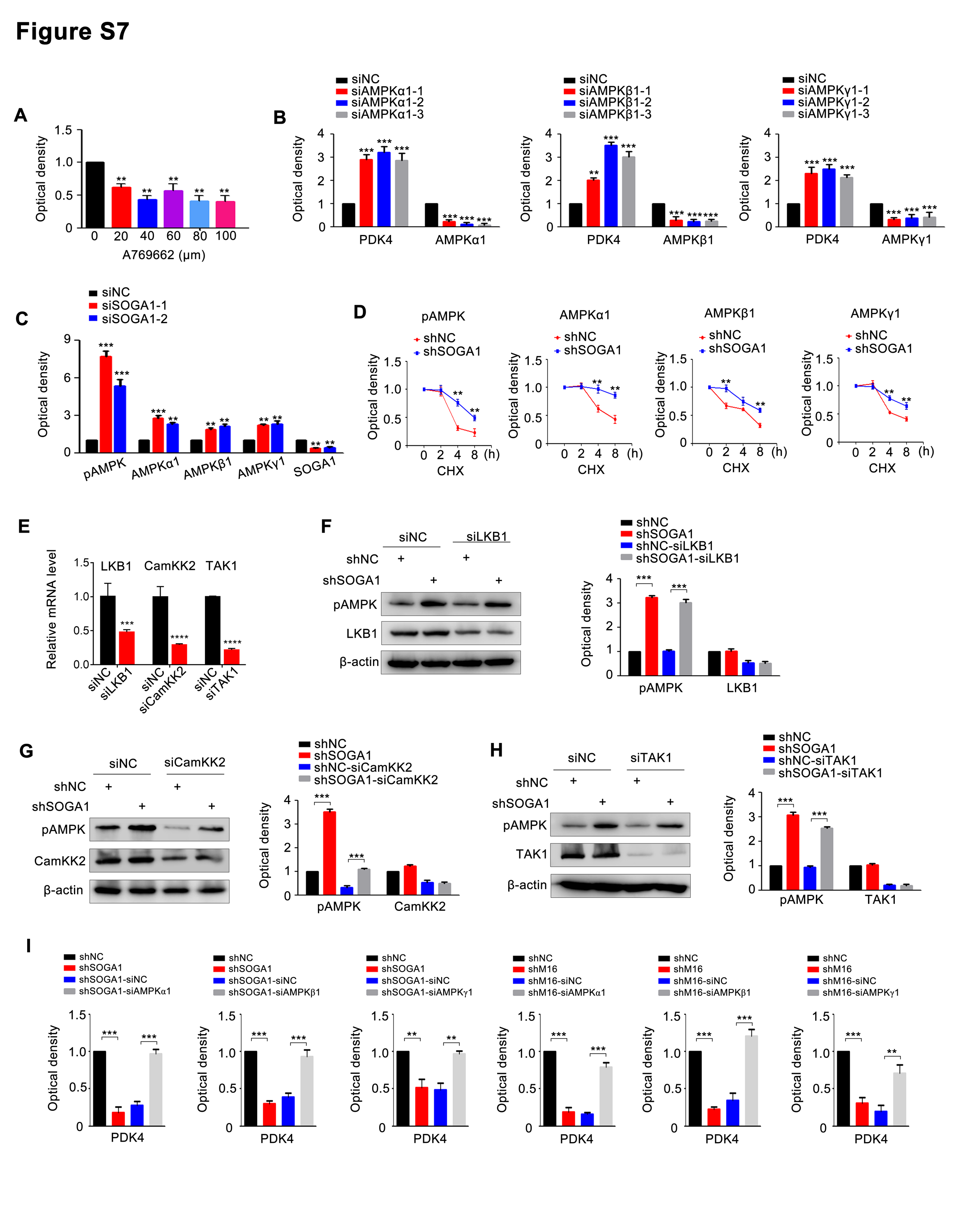


**Figure S7**. (A) The quantitatively analysis of western blotting results about the PDK4 protein expression in SW620 cells treated with different concentration of AMPK activator A769662. (B) The quantitatively analysis of western blotting results about the PDK4 protein expression in SW620 cells with or without AMPKα1, β1, γ1 knockdown. (C) The quantitatively analysis of western blotting results about the AMPKα1, β1, γ1 protein expression in SW620 cells with or without SOGA1 knockdown. (D) The quantitatively analysis of western blotting results about the protein stability of pAMPK, AMPKα1, β1, γ1 in SOGA1-konckdown and control cells at the indicated time after CHX (100 μg/ml) treatment. (E) The knockdown efficiency of LKB1, CaMKK2, and TAK1 in SW620 cells was detected by qRT-PCR respectively. (F-H) The effects of inhibition of LKB1, CaMKK2, and TAK1 on pAMPK expression in SOGA1 deficient SW620 cells were detected by western blotting. (I) The quantitatively analysis of western blotting results about the PDK4 protein expressions in SOGA1 or METTL16 deficient SW620 cell transfected with siRNAs of AMPKα1, β1, γ1 respectively. **P<0.01, ***P<0.001, ****P<0.0001.


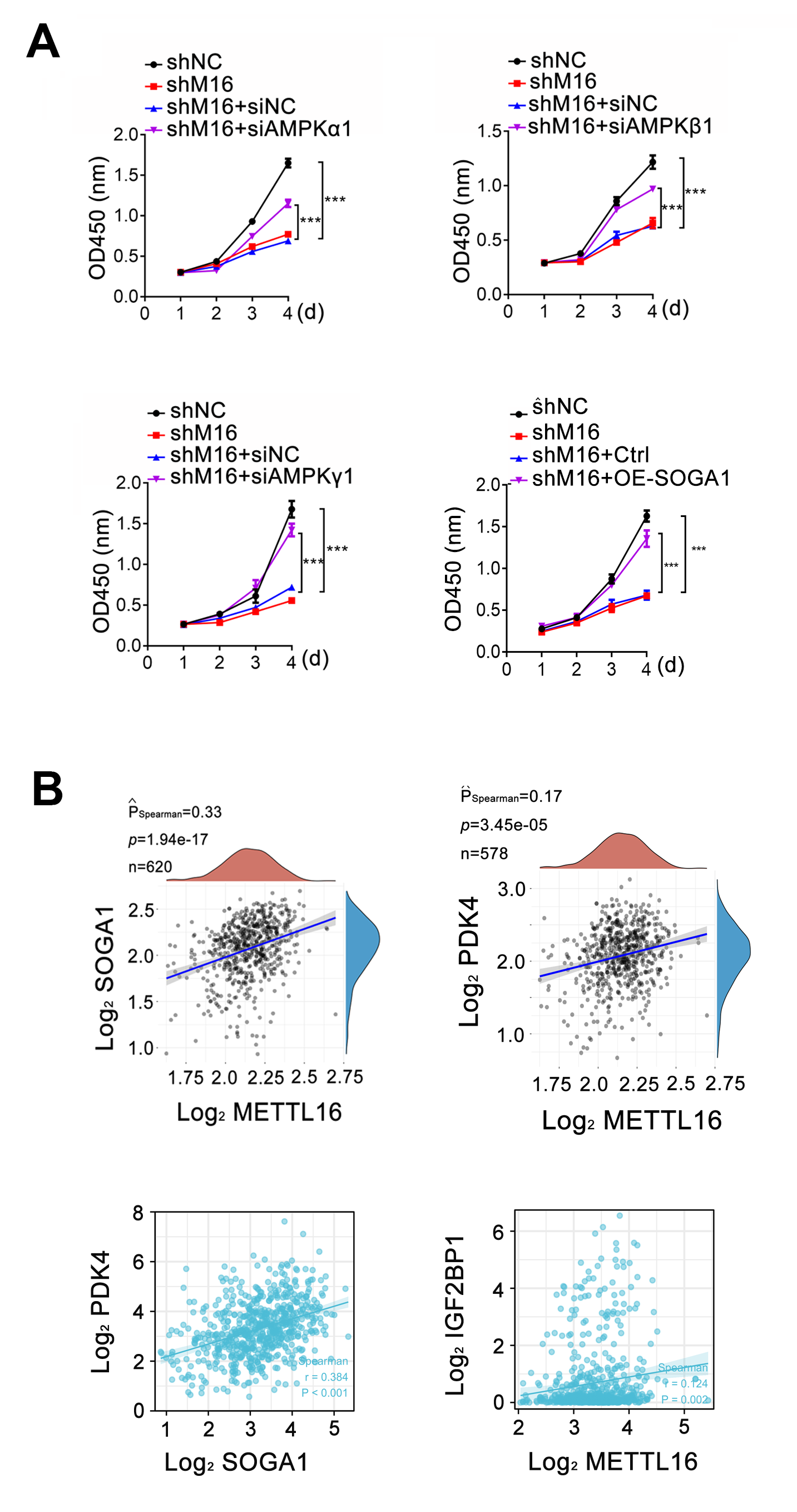


**Figure S8.** (A) CCK8 assay was conducted to measure proliferation of SW620-shM16 cells transfected with siAMPKα1, β1, γ1, and SOGA1 overexpression vector, respectively. (B) Correlation analysis between the expression of METTL16 and SOGA1, METTL16 and PDK4, SOGA1 and PDK4, METTL16 and IGF2BP1 based on TCGA database.
