# Supplementary material for: METTL16 promotes glycolytic metabolism reprogramming and colorectal cancer progression
Source: J Exp Clin Cancer Res. 2023 Jun 20;42:151. doi: 10.1186/s13046-023-02732-y (PMC10280857; doi:10.1186/s13046-023-02732-y)
Supplement: Supplementary file 2 — Additional file 2: Table S1. The sequences of siRNAs or shRNAs. [file 13046_2023_2732_MOESM2_ESM.docx]

| Oligonucleotides | Sequences |
| --- | --- |
| scrambled siRNA | UUCUCCGAACGUGUCACGUTT |
| siTCF4 | GCUCUGAGAUCAAAUCCGATT |
| siETS1 | CCGUGCUGACCUCAAUAAGTT |
| siYTHDF2 | CCAUGAUUGAUGGACAGUCAGCUUUTT |
| siXBP1 | GGAACAGCAAGUGGUAGAUTT |
| siVDR | GGAGUUCAUUCUGACAGAUTT |
| siYY1-1 | CCUGAAAUCUCACAUCUUATT |
| siYY1-2 | CAUCUUAACACAUGCUAAGGCCAAATT |
| siCEBPB | GAAACUUUAGCGAGUCAGATT |
| siYTHDF1-1 | CCUGCUCUUCAGCGUCAAUTT |
| siYTHDF1-2 | GGAACAACAUCUAUCAGCATT |
| siIGF2BP2-1 | CAUGCCGCAUGAUUCUUGATT |
| siIGF2BP2-2 | GAACGAACUGCAGAACUUATT |
| siIGF2BP1-1 | CCGGGAGCAGACCAGGCAATT |
| siIGF2BP1-2 | UGAAUGGCCACCAGUUGGATT |
| siIGF2BP3-1 | CGGUGAAUGAACUUCAGAATT |
| siIGF2BP3-2 | GCAAAGGAUUCGGAAACTT |
| siMETTL16-1 | AUGGCUGGUAUUUCCUCGCAATT |
| siMETTL16-2 | GGAAGAUUUUGGACUUUCUTT |
| siAMPKα1-1 | GGUUGGCAAACAUGAAUUGTT |
| siAMPKα1-2 | GAAGAUCGGCCACUACAUUTT |
| siAMPKα1-3 | GAGGAGAGCUAUUUGAUUATT |
| siAMPKβ1-1 | CCCAAGAUCCUGAUGGACATT |
| siAMPKβ1-2 | CACCAGAGAAGGAGGAAUUTT |
| siAMPKβ1-3 | UCUGGAAGUGAAUGAUAAATT |
| siAMPKγ1-1 | UGGGAUAGUAAGAAGCAAATT |
| siAMPKγ1-2 | GAAGAACACAAGAUAGAAATT |
| siAMPKγ1-3 | GCUCACAGGUGGAGAGAAGTT |
| siLKB1 | CCAACGUGAAGAAGGAAAUTT |
| siCamKK2 | GGAUCUGAUCAAAGGCAUCTT |
| siTAK1 | GGUGCUGAACCAUUGCCAUTT |
| shSOGA1-1 | GGAAATGGTTTAAAGAGAA |
| shSOGA1-2 | AGAAAGACACCAAGGAGAA |
| shMETTL16-1 | ATGGCTGGTATTTCCTCGCAA |
| shMETTL16-2 | GGAAGATTTTGGACTTTCT |

Table S1. The sequences of siRNAs or shRNAs.
