# Supplementary material for: METTL16 promotes glycolytic metabolism reprogramming and colorectal cancer progression
Source: J Exp Clin Cancer Res. 2023 Jun 20;42:151. doi: 10.1186/s13046-023-02732-y (PMC10280857; doi:10.1186/s13046-023-02732-y)
Supplement: Supplementary file 3 — Additional file 3: Table S2. Primers used in the study. [file 13046_2023_2732_MOESM3_ESM.docx]

| Table S2. Primers used in the study. | |
| --- | --- |
| Primers | Sequences（5'-3'） |
| METTL14 forward primer | AGGGAGTAAACTCACGAAATCCT |
| METTL14 reverse primer | AACCCCTTGTATGCGAAGCTC |
| XBP1 forward primer | CCTGGTTGCTGAAGAGGAGG |
| XBP1 reverse primer | CCATGGGGAGATGTTCTGGAG |
| VDR forward primer | AGCGGAAGGCACTATTCACC |
| VDR reverse primer | CATCATGCCGATGTCCACACA |
| EST1 forward primer | GATAGTTGTGATCGCCTCACC |
| EST1 reverse primer | GTCCTCTGAGTCGAAGCTGTC |
| YY1 forward primer | AGATCCCAAACAACTGGCAGA |
| YY1 reverse primer | TCTTTGTGCAGCCTTTATGAGG |
| TCF4 forward primer | GAAAGCTGCGTGTCTGAAAA |
| TCF4 reverse primer | CATCTGTCCCATGTGATTCG |
| CEBPB forward primer | CTTCAGCCCGTACCTGGAG |
| CEBPB reverse primer | GGAGAGGAAGTCGTGGTGC |
| GLUT1 forward primer | GGCCAAGAGTGTGCTAAAGAA |
| GLUT1 reverse primer | ACAGCGTTGATGCCAGACAG |
| GLUT2 forward primer | GCTGCTCAACTAATCACCATGC |
| GLUT2 reverse primer | TGGTCCCAATTTTGAAAACCCC |
| GLUT4 forward primer | TGGGCGGCATGATTTCCTC |
| GLUT4 reverse primer | GCCAGGACATTGTTGACCAG |
| GLUT10 forward primer | CTTGCTGTATCTACGTGTCAGAG |
| GLUT10 reverse primer | CCAGCCAGTGCATAGTTGAGG |
| PKM2 forward primer | ATGTCGAAGCCCCATAGTGAA |
| PKM2 reverse primer | TGGGTGGTGAATCAATGTCCA |
| HK2 forward primer | GAGCCACCACTCACCCTACT |
| HK2 reverse primer | CCAGGCATTCGGCAATGTG |
| HK3 forward primer | GGACAGGAGCACCCTCATTTC |
| HK3 reverse primer | CCTCCGAATGGCATCTCTCAG |
| PDK1 forward primer | CTGTGATACGGATCAGAAACCG |
| PDK1 reverse primer | TCCACCAAACAATAAAGAGTGCT |
| PDK2 forward primer | ATGAAAGAGATCAACCTGCTTCC |
| PDK2 reverse primer | GGCTCTGGACATACCAGCTC |
| PDK3 forward primer | CGCTCTCCATCAAACAATTCCT |
| PDK3 reverse primer | CCACTGAAGGGCGGTTAAGTA |
| PDK4 forward primer | GGAGCATTTCTCGCGCTACA |
| PDK4 reverse primer | ACAGGCAATTCTTGTCGCAAA |
| ENO1 forward primer | AAAGCTGGTGCCGTTGAGAA |
| ENO1 reverse primer | GGTTGTGGTAAACCTCTGCTC |
| ENO2 forward primer | AGCCTCTACGGGCATCTATGA |
| ENO2 reverse primer | TTCTCAGTCCCATCCAACTCC |
| ENO3 forward primer | GGCTGGTTACCCAGACAAGG |
| ENO3 reverse primer | TCGTACTTCCCATTGCGATAGAA |
| AMPKα1 forward primer | TTGAAACCTGAAAATGTCCTGCT |
| AMPKα1 reverse primer | GGTGAGCCACAACTTGTTCTT |
| AMPKβ1 forward primer | CCACTCCGAGGAAATCAAGGC |
| AMPKβ1 reverse primer | CTGGGCGGGAGCTTTATCA |
| AMPKγ1 forward primer | ATGAAGTCTCATCGCTGCTATG |
| AMPKγ1 reverse primer | ACCGTTAGTCACCAAAGCAAA |
| LKB1 forward primer | TCTACAACATCACCACGGGTC |
| LKB1 reverse primer | TTCGTACTCAAGCATCCCTTTC |
| CamKK2 forward primer | AAGACCAGGCCCGTTTCTACT |
| CamKK2 reverse primer | CCAGGAGGTTGGAAGGTTTGA |
| TAK1 forward primer | CCGGTGAGATGATCGAAGCC |
| TAK1 reverse primer | GCCGAAGCTCTACAATAAACGC |
| IGF2BP1 forward primer | TAGTACCAAGAGACCAGACCC |
| IGF2BP1 reverse primer | GATTTCTGCCCGTTGTTGTC |
| LRG1 forward primer | GGACACCCTGGTATTGAAAGAAA |
| LRG1 reverse primer | TAGCCGTTCTAATTGCAGCGG |
| HOXA3 forward primer | ATGCAAAAAGCGACCTACTACG |
| HOXA3 reverse primer | TACGGCTGCTGATTGGCATTA |
| EID3 forward primer | GCCGACGTAGACCCAAAGC |
| EID3 reverse primer | GTTAAGGAGTTGTTCGCCGAG |
| PAQR6 forward primer | CCTTCCCCTATGCCGCCTA |
| PAQR6 reverse primer | GGAGGACCTTACTGAGCCC |
| B3GNT4 forward primer | ACACAGTGTCTAGCGCCTCT |
| B3GNT4 reverse primer | AAGGTATCCTTGGAACAGCCT |
| ZNF778 forward primer | GGCTGGCTGATAAATTGTTACCA |
| ZNF778 reverse primer | GTAGAGGTCTCTCTGAGATGGG |
| SOGA1 forward primer | GAGCAGGATGTCAAGGTCTCT |
| SOGA1reverse primer | TTAGCCAGCTCAGTCTCGATG |
| β-actin forward primer | CATGTACGTTGCTATCCAGGC |
| β-actin reverse primer | CTCCTTAATGTCACGCACGAT |
| GAPDH forward primer | GGAGCGAGATCCCTCCAAAAT |
| GAPDH reverse primer | GGCTGTTGTCATACTTCTCATGG |
|  |  |
| SOGA1 probe | AGCAGGAAGTTGTGCTTGAATTGCT |
| control-probe | AGCAATTCAAGCACAACTTCCTGCT |
|  |  |
| ChIPprimer1 forward primer | TTTTCTCGCGAATTCGAACGC |
| ChIPprimer1 reverse primer | AGAAGAAACCCTCGCGACAC |
| ChIPprimer2 forward primer | TGAGGACACACCTGTTGCAC |
| ChIPprimer2 reverse primer | AACAAGTTTTCCGGGCCCT |
