# Supplementary material for: METTL16 promotes glycolytic metabolism reprogramming and colorectal cancer progression
Source: J Exp Clin Cancer Res. 2023 Jun 20;42:151. doi: 10.1186/s13046-023-02732-y (PMC10280857; doi:10.1186/s13046-023-02732-y)
Supplement: Supplementary file 4 — Additional file 4: Table S3. METTL16 expression in clinical and pathologicalcharacteristics of colorectal cancer patients. [file 13046_2023_2732_MOESM4_ESM.docx]

| **Variable** | **Expression of METTL16 in colorectal cancer** | | | ***P* value** |
| --- | --- | --- | --- | --- |
|  | **High**  **（n=36）** | **Low**  **（n=50）** | **Total**  **(n=86)** |  |
| **Age** |  |  |  |  |
| <55 | 12 | 17 | 29 | 0.949 |
| ≥55 | 24 | 33 | 57 |  |
| **Gender** |  |  |  |  |
| Male | 25 | 29 | 54 | 0.279 |
| Female | 11 | 21 | 32 |  |
| **Tumor size** |  |  |  |  |
| ≤4 | 17 | 23 | 40 | 0.911 |
| >4 | 19 | 27 | 46 |  |
| **Histological grade** |  |  |  |  |
| Ⅰ-Ⅱ | 29 | 40 | 69 | 0.949 |
| Ⅱ-Ⅲ | 7 | 10 | 17 |  |
| **Lymph node** |  |  |  |  |
| Positive | 15 | 26 | 41 | 0.344 |
| Negative | 21 | 24 | 45 |  |
| **Tumor stage** |  |  |  |  |
| T1-T3 | 15 | 29 | 44 | 0.135 |
| T4 | 21 | 21 | 42 |  |

Table S3. METTL16 expression in clinical and pathological characteristics of colorectal cancer patients.
