# Supplementary figures and images for: METTL16 promotes glycolytic metabolism reprogramming and colorectal cancer progression
Source: J Exp Clin Cancer Res. 2023 Jun 20;42:151. doi: 10.1186/s13046-023-02732-y (PMC10280857; doi:10.1186/s13046-023-02732-y)

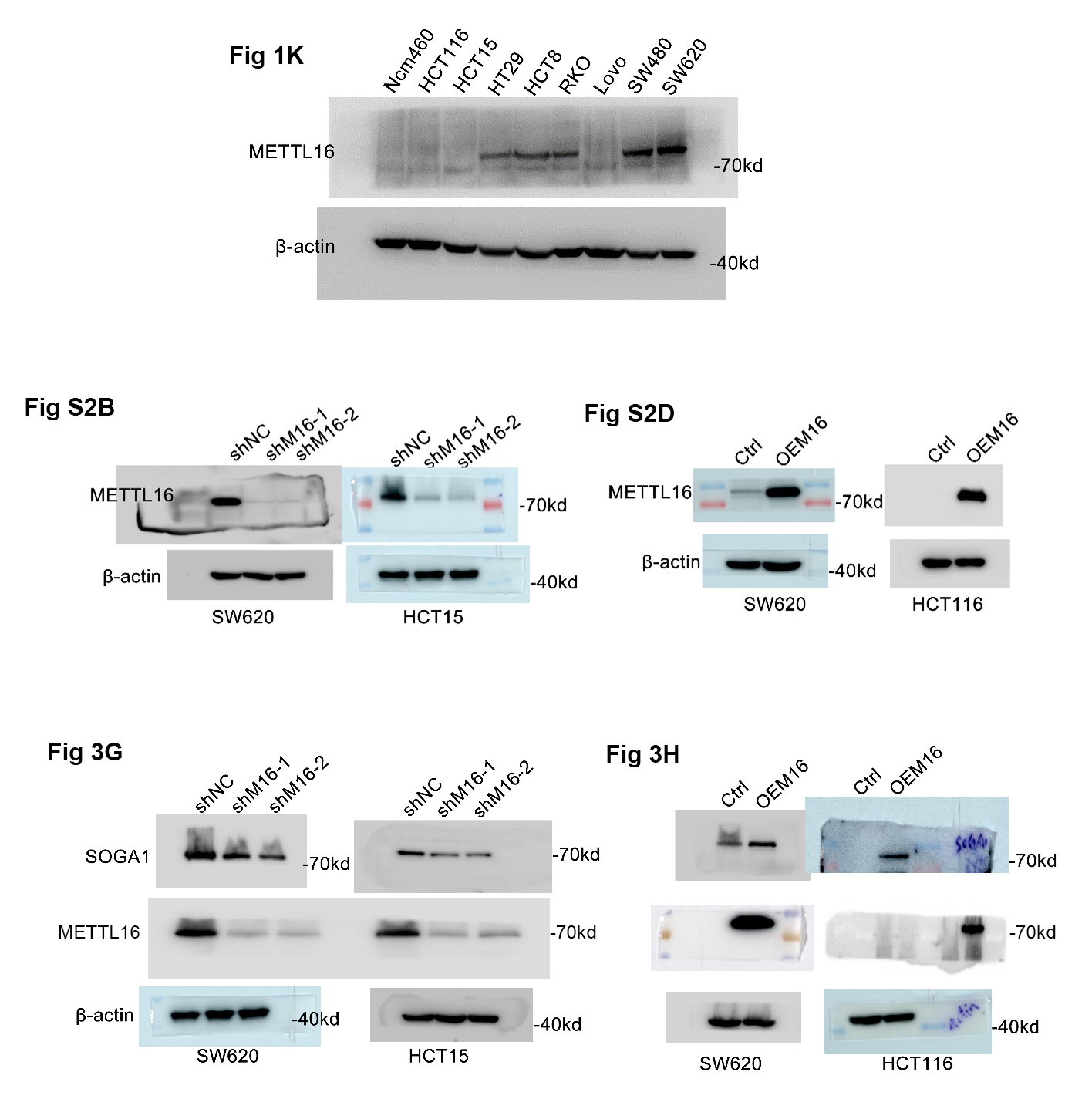


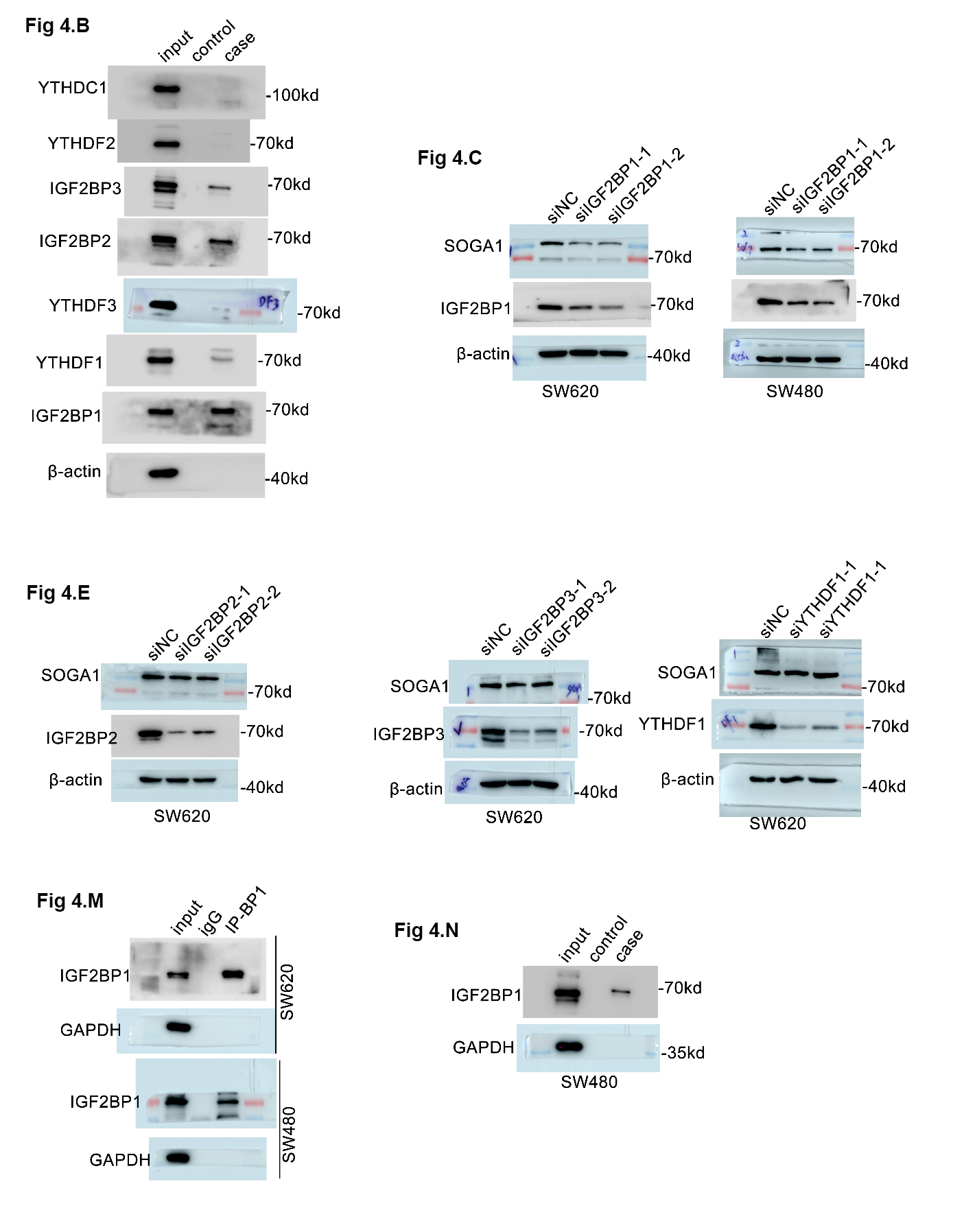


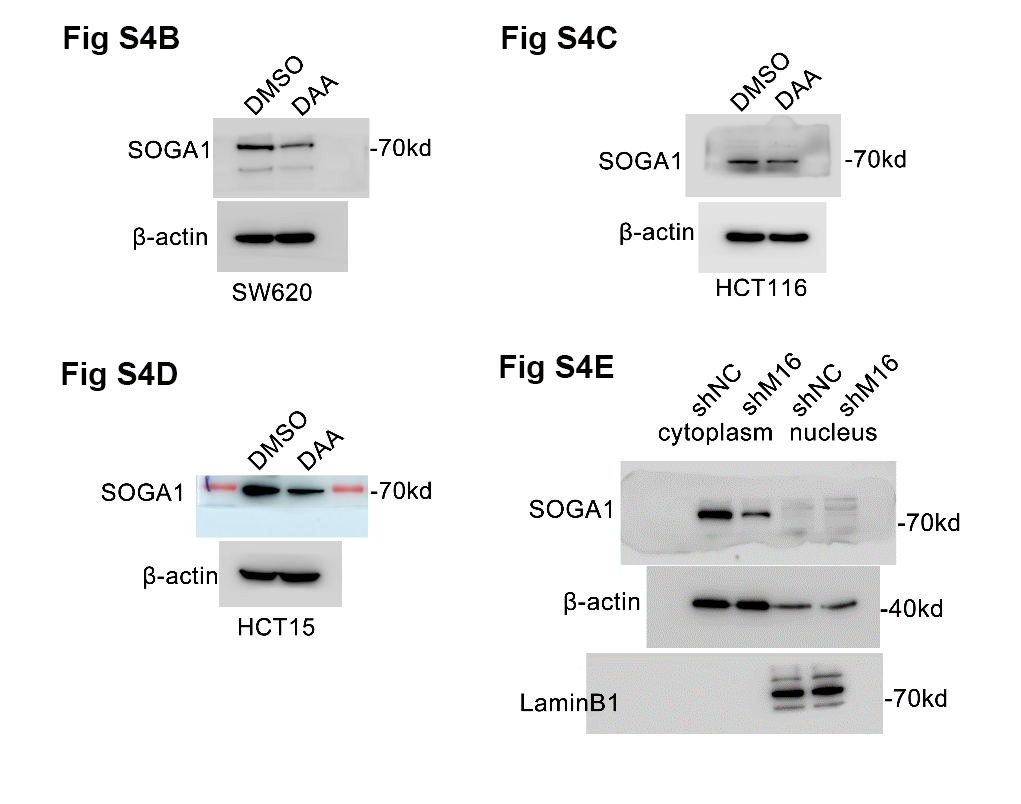


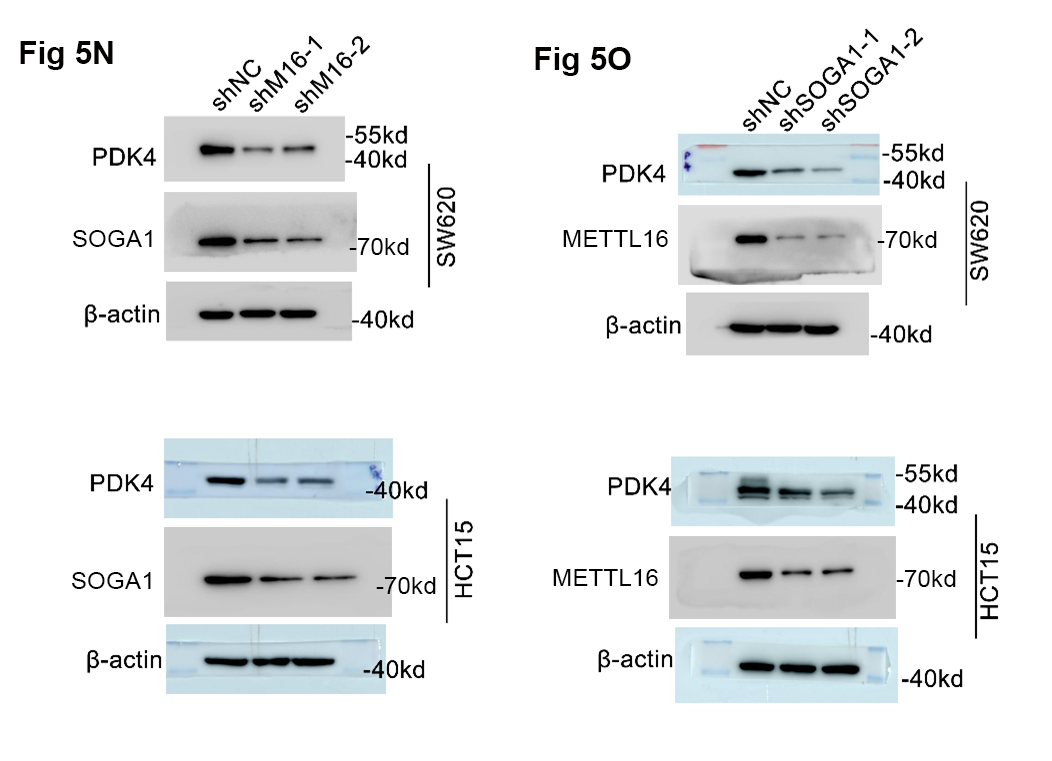


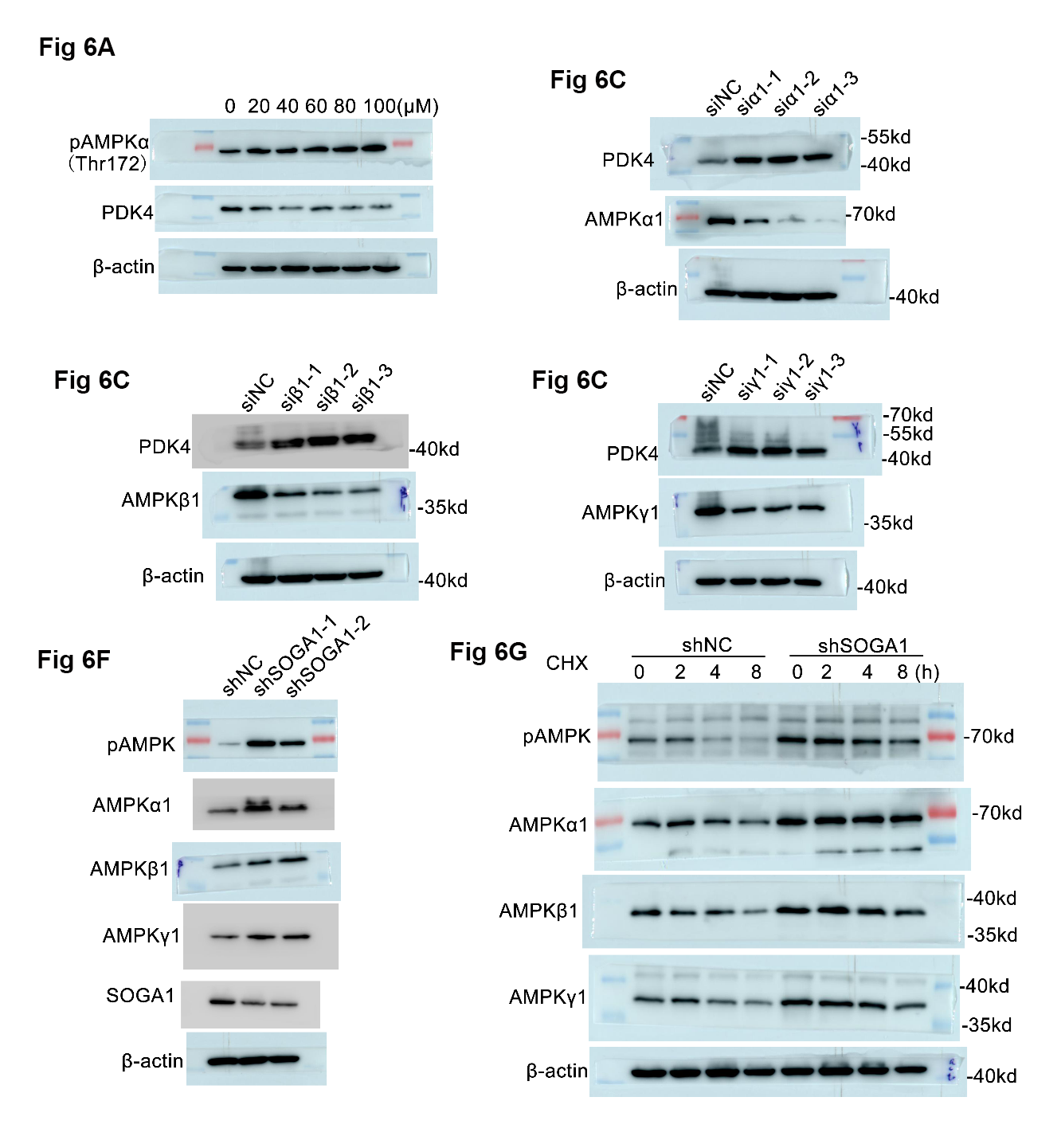


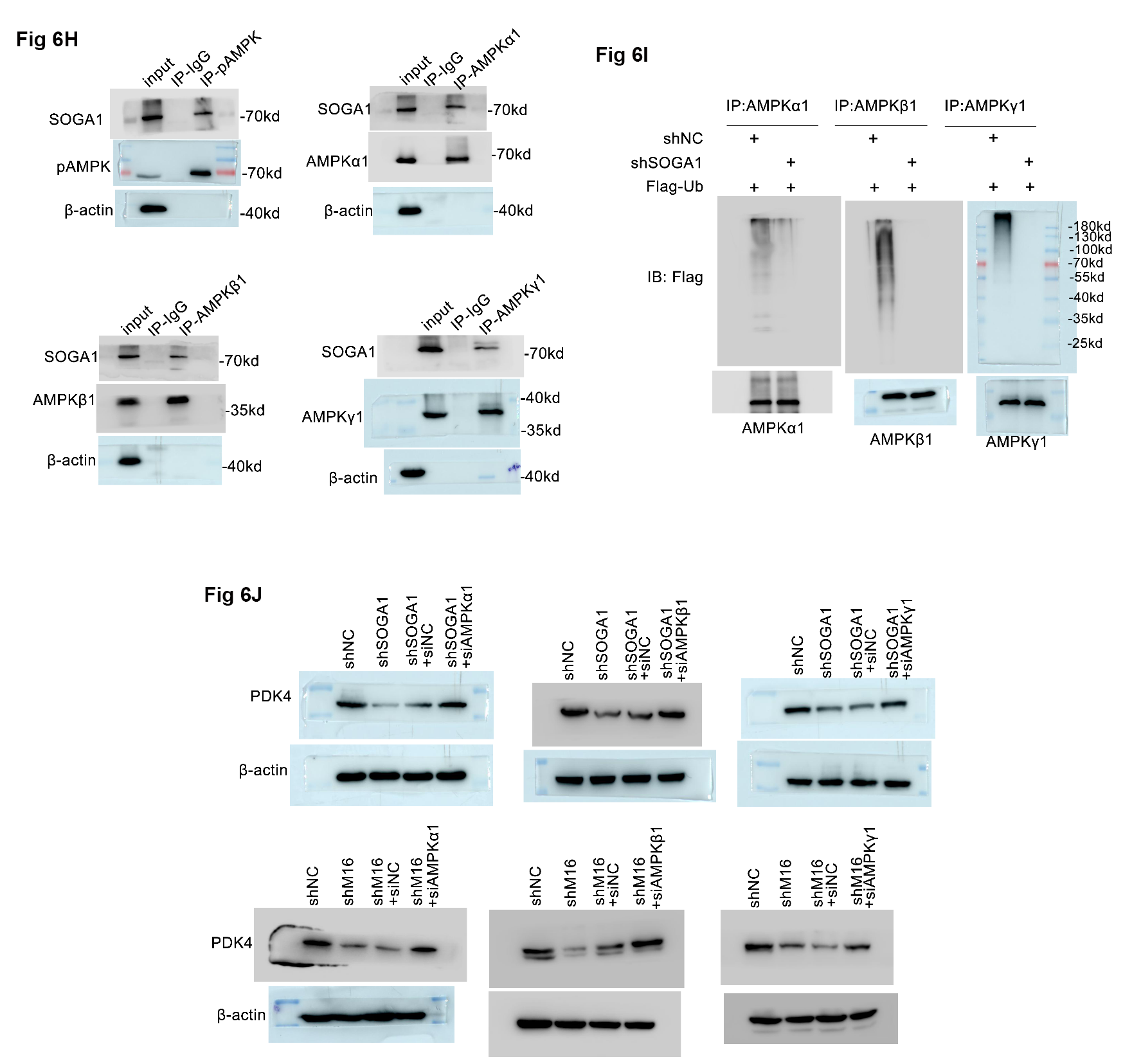


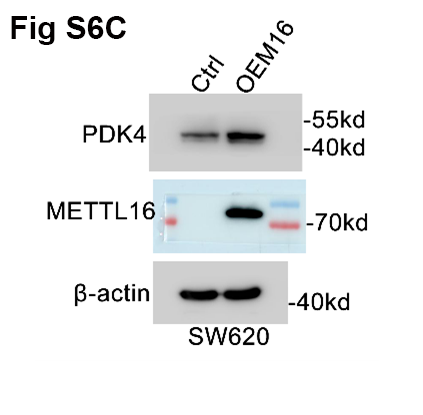


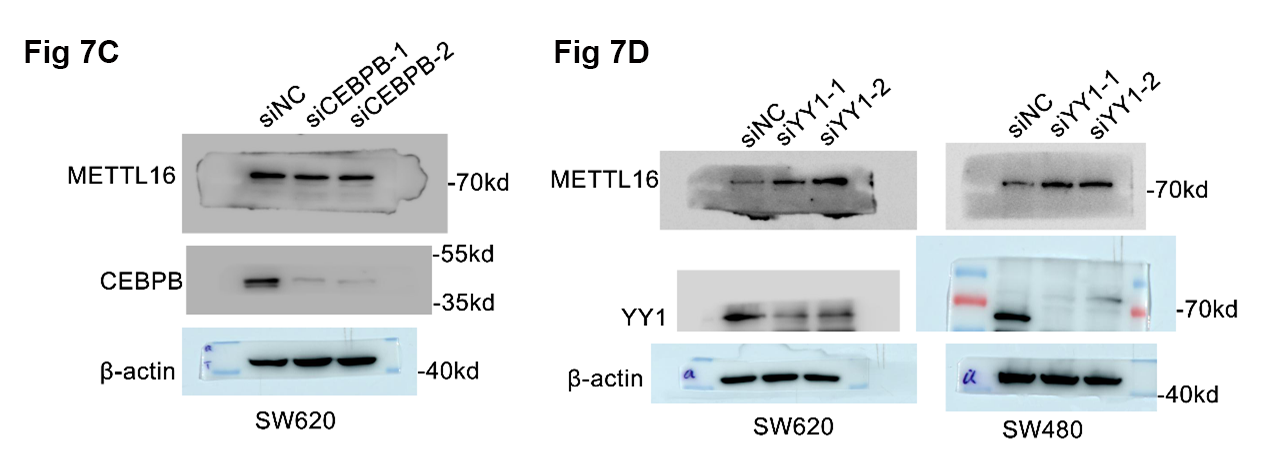


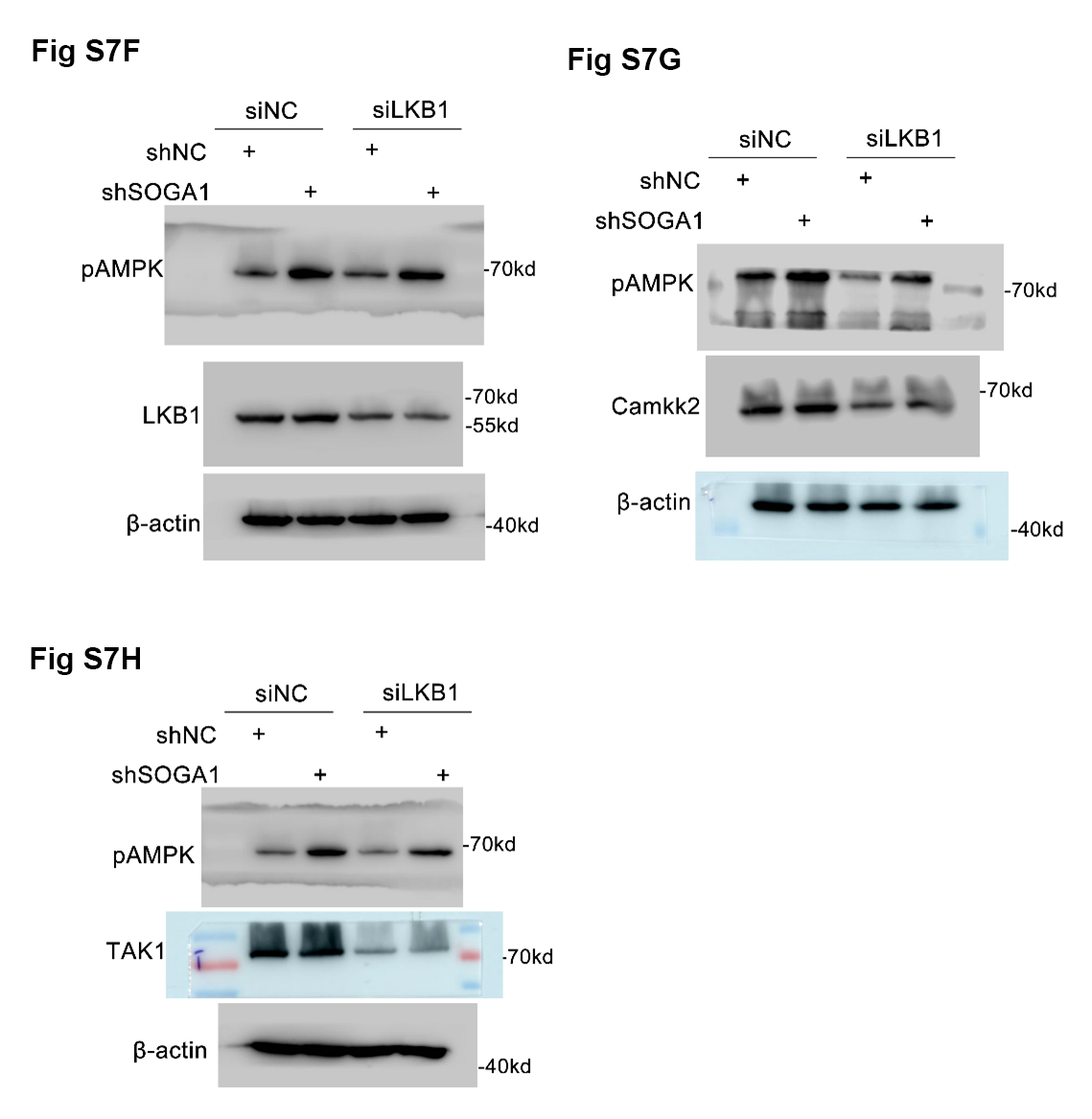

Supplement: Supplementary file 5 — Additional file 5. [file 13046_2023_2732_MOESM5_ESM.docx]
